# Supplementary material for: Geology controls the distribution of a seed-eating bird: Feeding-tree selection by the glossy black-cockatoo Calyptorhynchus lathami
Source: PLoS One. 2024 Aug 8;19(8):e0308323. doi: 10.1371/journal.pone.0308323 (PMC11309512; doi:10.1371/journal.pone.0308323)
Supplement: S1 Table — Status correct as at November 20, 2023. (PDF) [file pone.0308323.s001.pdf]

S1 Table. Conservation status of the glossy black-cockatoo.

Status correct as at November 20, 2023.

			Threats									
**Taxon**	**Jurisdiction**	**Status**	**Feeding habitat loss and degradation**	**Loss of/competition for nest sites**	**Predation by common brush-tailed possums**	**Lack of watering points**	**Adverse fire regime**	**Drought/climate change**	**Bird and/or egg collection**	**Grazing of regenerating habitat**	**Disease**	**References**
Calyptorhynchus lathami	International	Vulnerable	Yes	Yes	Yes			Yes				[1]
	New South Wales	Vulnerable	Yes	Yes		Yes	Yes	Yes	Yes	Yes		[2, 3]
	Australian Capital Territory	Vulnerable	Yes	Yes	Possible		Yes	Yes	Possible			[4]
	Queensland	Vulnerable										[5]
	Victoria	Vulnerable	Yes	Yes			Yes	Yes				[6]
Calyptorhynchus lathami lathami	Australia	Vulnerable	Yes	Yes	Yes	Possible	Yes	Yes	Possible		Yes	[7, 8]
		Near threatened	Yes	Yes	Yes		Yes			Yes		[9]
Calyptorhynchus lathami halmaturinus	Australia	Endangered	Yes	Yes	Yes		Yes		Possible		Possible	[8, 10, 11]
	South Australia	Endangered	Yes	Yes	Yes		Yes	Yes			Yes	[12]

References

1. BirdLife International. Species factsheet: *Calyptorhynchus lathami* Data Zone [Internet]. 2023 [cited 2023 November 20]. Available from: http://datazone.birdlife.org/species/factsheet/glossy-black-cockatoo-calyptorhynchus-lathami/details

2. Office of Environment and Heritage. South-eastern Glossy Black-Cockatoo. Threatened Biodiversity Profile [Internet]. 2023 [cited 2023 November 20]. Available from: https://threatenedspecies.bionet.nsw.gov.au/profile?id=10140.
3. New South Wales Government. Glossy black-cockatoo. Native Animal Facts: Parrots [Internet]. 2022 [cited 2023 November 20]. Available from: https://www.environment.nsw.gov.au/topics/animals-and-plants/native-animals/native-animal-facts/parrots/glossy-black-cockatoo.
4. Environment, Planning and Sustainable Development Directorate. Glossy Black-Cockatoo (*Calyptorhynchus lathami*) Environment [Internet]. 2023 [cited 2023 November 20]. Available from: https://www.environment.act.gov.au/files/documents/nature-conservation/factsheets2/glossy_black-cockatoo.
5. Queensland Government. *Calyptorhynchus lathami* (glossy black-cockatoo). Species Profile [Internet]. 2022 25 August 2023 [cited 2023 November 20]. Available from: https://apps.des.qld.gov.au/species-search/details/?id=1171.
6. Department of Environment and Primary Industries. Action Statement No. 253. Glossy Black-Cockatoo *Calyptorhynchus lathami*. Melbourne: State Government of Victoria; 2013 [cited 2023 November 20]. Available from: https://www.environment.vic.gov.au/__data/assets/pdf_file/0013/32305/Glossy_Black_Cockatoo_Calyptorhynchus_lathami.pdf.
7. Department of Climate Change, Energy, the Environment and Water. *Calyptorhynchus lathami lathami* — South-eastern Glossy Black-Cockatoo. Species Profile and Threats Database [Internet]. 2022 [cited 2023 November 20]. Available from: http://www.environment.gov.au/cgi-bin/sprat/public/publicspecies.pl?taxon_id=67036.
8. Wildlife and Threatened Species Bushfire Recovery Expert Panel. Provisional list of animals requiring urgent management intervention. Canberra: Department of Agriculture, Water and the Environment; 2020 [cited 2023 November 20]. Available from: www.environment.gov.au/system/files/pages/ef3f5ebd-faec-4c0c-9ea9-b7dfd9446cb1/files/assessments-species-vulnerability-fire-impacts-14032020.pdf.
9. Cameron M, Castley G, Teixeira D, Menkhorst PW, Garnett ST. South-eastern Glossy Black-Cockatoo *Calyptorhynchus lathami lathami* Temminck, 1807. In: Garnett ST, Baker GB, editors. The Action Plan for Australian Birds 2020. Melbourne: CSIRO Publishing; 2021. p. 395-8.
10. Department of Climate Change, Energy, the Environment and Water. *Calyptorhynchus lathami halmaturinus* — Kangaroo Island Glossy Black-Cockatoo, Glossy Black-Cockatoo (South Australian). Species Profile and Threats Database [Internet]. 2022 [cited 2023 November 20]. Available from: http://www.environment.gov.au/cgi-bin/sprat/public/publicspecies.pl?taxon_id=64436.
11. Berris KK, Mooney PA, Barth M, Welz T, Crowley GM. Kangaroo Island Glossy Black-Cockatoo *Calyptorhynchus lathami halmaturinus*. In: Garnett ST, Baker GB, editors. The Action Plan for Australian Birds 2020. Melbourne: CSIRO Publishing; 2021. p. 391-4.
12. National Parks and Wildlife Act 1972, Schedule 7 - Endangered Species NPW Act 1972 (2023).
