# Supplementary material for: Geology controls the distribution of a seed-eating bird: Feeding-tree selection by the glossy black-cockatoo Calyptorhynchus lathami
Source: PLoS One. 2024 Aug 8;19(8):e0308323. doi: 10.1371/journal.pone.0308323 (PMC11309512; doi:10.1371/journal.pone.0308323)
Supplement: S2 Table — r values are displayed above the diagonal. P values are displayed below the diagonal. (PDF) [file pone.0308323.s002.pdf]

S2 Table. Correlation matrix for Seed Fill and Kernel Ratio.

r values are displayed above the diagonal. P values are displayed below the diagonal.

	Seed Fill	log (Seed Fill)	Kernel Ratio	log (Kernel Ratio)
**Seed Fill**			0.112	0.109
**log (Seed Fill)**			0.060	0.055
**Kernel Ratio**	0.3336	0.6057		
**log (Kernel Ratio)**	0.3467	0.6359		
