# Supplementary material for: Geology controls the distribution of a seed-eating bird: Feeding-tree selection by the glossy black-cockatoo Calyptorhynchus lathami
Source: PLoS One. 2024 Aug 8;19(8):e0308323. doi: 10.1371/journal.pone.0308323 (PMC11309512; doi:10.1371/journal.pone.0308323)
Supplement: S3 Table — FV = Food value; SF = Seed Fill; KR = Kernel ratio. Robust regression modelling was undertaken using the lmrob function in the R package robustbase. (PDF) [file pone.0308323.s003.pdf]

S3 Table. Robust regression models explaining the influence of Seed Fill and Kernel Ratio on Food Value.

FV = Food value; SF = Seed Fill; KR = Kernel ratio. Robust regression modelling was undertaken using the lmrob function in robustbase [1].

**Equation**	**FV ~ SF + KR**		**FV ~ log(SF) + KR**		**FV ~ SF + log(KR)**		**FV ~ log(SF) + log(KR)**	
**Variable**	**Estimate**	**P**	**Estimate**	**P**	**Estimate**	**P**	**Estimate**	**P**
Intercept	-11.599	0.009	21.488	<0.0001	9.7193	0.004	46.404	<0.0001
SF	40.624	<0.0001	-	-	40.623	<0.0001	-	-
log(SF)	-	-	16.639	<0.0001	-	-	16.632	<0.0001
KR	26.021	0.004	30.718	0.005	-	-	-	-
log(KR)	-	-	-	-	11.629	0.002	13.438	0.001
**Statistic**								
Robust residual standard error	4.684		5.566		4.669		5.526	
Multiple R^2	0.6863		0.577		0.6932		0.5904	
Adjusted R^2	0.6779		0.5656		0.6849		0.5793	
No. iterations	14		14		14		12	

Reference

1. Maechler M, Rousseeuw P, Croux C, Todorov V, Ruckstuhl A, Salibian-Barrera M, et al. robustbase: Basic Robust Statistics. Version 0.99-1. 2023.
