# Supplementary material for: Geology controls the distribution of a seed-eating bird: Feeding-tree selection by the glossy black-cockatoo Calyptorhynchus lathami
Source: PLoS One. 2024 Aug 8;19(8):e0308323. doi: 10.1371/journal.pone.0308323 (PMC11309512; doi:10.1371/journal.pone.0308323)
Supplement: S4 Table — (PDF) [file pone.0308323.s004.pdf]

S4 Table. Processing of black sheoak and forest oak records and glossy black-cockatoo feeding records.

Criteria	Black sheoak	Forest oak	Feeding records
**Rejection**			
Records from outside NSW.	Reject	Reject	Reject
Records with accuracy > 2km.	Reject	Reject	Reject
Records marked as "N" (i.e. not located).	Reject	Reject	Reject
Records of plants growing in cultivation.	Reject	Reject	Reject
Records with a “First date” of 1 Jan 1900, assuming this was the default value when no date was provided.	Reject	Reject	Reject
All but one record of duplicate records having:			
• exactly the same “Lat”, “Long” (or location description) and “Last date”.	Reject	Reject	Reject
• exactly the same “Lat”, “Long” (or location description) on consecutive days.	Reject	Reject	Reject
**Acceptance**			
Records of either *Allocasuarina littoralis* (or “black sheoak”) or *Allocasuarina torulosa* (or “forest oak”) in which:			
• “crushed cones” were recorded in the “Observation type” field.			Accept
• “feeding” was recorded in the “Sighting notes” field.			Accept
• “Feeding sign recognition” was recorded in the “Sighting notes” field.			Accept
**Jittering**			
To protect this threatened species, location of all grid cells was obscured by setting the minimum easting and northing values to zero, and adding a random distance of between 100 m and 1,000 m to each resultant easting and northing.	Jitter	Jitter	Jitter
