# Supplementary material for: Geology controls the distribution of a seed-eating bird: Feeding-tree selection by the glossy black-cockatoo Calyptorhynchus lathami
Source: PLoS One. 2024 Aug 8;19(8):e0308323. doi: 10.1371/journal.pone.0308323 (PMC11309512; doi:10.1371/journal.pone.0308323)
Supplement: S5 Table — Classification based on rock unit descriptions from Raymond OL, Liu S, Gallagher R, Zhang W, Highet LM. Surface Geology of Australia 1:1 Million Scale Dataset. 2012 Edition. Canberra: Geoscience Australia; 2012. (PDF) [file pone.0308323.s005.pdf]

S5 Table. Classification of New South Wales rock types into sedimentary rocks, calcareous sedimentary rocks and other rock types.

Classification based on rock unit descriptions from Raymond OL, Liu S, Gallagher R, Zhang W, Highet LM. Surface Geology of Australia 1:1 Million Scale Dataset. 2012 Edition. Canberra: Geoscience Australia; 2012

Rock unit	Sedimentary	Calcareous	Rock type
Adaminaby Group	Yes	No	Non-calcareous sedimentary rock
Agnes Greywacke	Yes	No	Non-calcareous sedimentary rock
Allandale Formation	Yes	No	Non-calcareous sedimentary rock
Alluvium 38485	No	No	Other rock type
Alluvium 38494	No	No	Other rock type
Allyn River Member	Yes	No	Non-calcareous sedimentary rock
Alum Mountain Volcanics	Yes	No	Non-calcareous sedimentary rock
Ararat Formation	Yes	Yes	Calcareous sedimentary rock
Argyll Granodiorite	No	No	Other rock type
Ballallaba Monzogranite	No	No	Other rock type
Beechwood beds	Yes	No	Non-calcareous sedimentary rock
Bellbird Creek Formation	Yes	No	Non-calcareous sedimentary rock
Bells Creek Volcanics	No	No	Other rock type
Belowra Granodiorite	No	No	Other rock type
Bemboka Granodiorite	No	No	Other rock type
Ben Boyd Formation	Yes	No	Non-calcareous sedimentary rock
Bendoc Group	Yes	No	Non-calcareous sedimentary rock
Bergalia Formation	No	No	Other rock type
Big Hole Formation	Yes	No	Non-calcareous sedimentary rock
Big Jack Granite	No	No	Other rock type
Billyrimba Leucomonzogranite	No	No	Other rock type
Billys Creek Tonalite	No	No	Other rock type
Bindook Group	No	No	Other rock type
Bingie Bingie Gabbro	No	No	Other rock type
Birdwood Formation	Yes	No	Non-calcareous sedimentary rock
Bitter Ground Volcanics	No	No	Other rock type
Blaxland Granite	No	No	Other rock type
Bodalla Monzogranite	No	No	Other rock type
Bogolo Formation	Yes	No	Non-calcareous sedimentary rock
Bolivia Range Leucomonzogranite	No	No	Other rock type
Bondonga beds	Yes	No	Non-calcareous sedimentary rock

Rock unit	Sedimentary	Calcareous	Rock type
Bonnington Siltstone	Yes	No	Non-calcareous sedimentary rock
Boolambayte Formation	Yes	Yes	Calcareous sedimentary rock
Boonanghi beds	Yes	No	Non-calcareous sedimentary rock
Booral Formation, Buckets Gap Formation	Yes	No	Non-calcareous sedimentary rock
Booti Booti Sandstone	Yes	No	Non-calcareous sedimentary rock
Boro Granite	No	No	Other rock type
Botumburra Range Monzogranite	No	No	Other rock type
Bowman beds	Yes	No	Non-calcareous sedimentary rock
Boyd Volcanic Complex	No	No	Other rock type
Braidwood Granodiorite	No	No	Other rock type
Branxton Formation	Yes	No	Non-calcareous sedimentary rock
Breckin Ignimbrite Member	No	No	Other rock type
Brogo Granodiorite	No	No	Other rock type
Brooklana beds	Yes	No	Non-calcareous sedimentary rock
Bruxner Monzogranite	No	No	Other rock type
Buckleys Lake Monzogranite	No	No	Other rock type
Buffers Creek Formation	Yes	No	Non-calcareous sedimentary rock
Bulahdelah Formation	Yes	No	Non-calcareous sedimentary rock
Bundamba Group	Yes	No	Non-calcareous sedimentary rock
Bundook beds	Yes	Yes	Calcareous sedimentary rock
Bundundah Granite	No	No	Other rock type
Bunga beds	Yes	No	Non-calcareous sedimentary rock
Bungonia Group	Yes	Yes	Calcareous sedimentary rock
Bungulla Monzogranite	No	No	Other rock type
Butmaroo Granite	No	No	Other rock type
Camden Haven Group	Yes	No	Non-calcareous sedimentary rock
Candelo Tonalite	No	No	Other rock type
Carrai Granodiorite	No	No	Other rock type
Chaelundi Complex	No	No	Other rock type
Chillingham Volcanics	No	No	Other rock type
coastal dunes 38488	No	No	Other rock type
Cobargo Granodiorite	No	No	Other rock type
Coffs Harbour Association	Yes	No	Non-calcareous sedimentary rock

Rock unit	Sedimentary	Calcareous	Rock type
Colinton Volcanics	No	No	Other rock type
colluvium 38491	No	No	Other rock type
Colraine Mudstone	Yes	No	Non-calcareous sedimentary rock
Comboyne Basalt	No	No	Other rock type
Combyingbar Formation	Yes	No	Non-calcareous sedimentary rock
Comerong Volcanics	No	No	Other rock type
Coogal Subgroup, Nea Subgroup	Yes	No	Non-calcareous sedimentary rock
Coolangubra Monzogranite	No	No	Other rock type
Copeland Road Formation, Faulkland Formation, Karuah Formation	Yes	No	Non-calcareous sedimentary rock
Coramba beds	Yes	No	Non-calcareous sedimentary rock
Cordeaux Crinanite	No	No	Other rock type
Cottesbrook Monzogranite	No	No	Other rock type
Cowangara Formation	Yes	No	Non-calcareous sedimentary rock
Crescent Complex	No	No	Other rock type
Crescent Head Formation	Yes	No	Non-calcareous sedimentary rock
Croajingalong Granite	No	No	Other rock type
Crudine Group	Yes	No	Non-calcareous sedimentary rock
Cunglebung Creek beds	Yes	No	Non-calcareous sedimentary rock
Currabubula Formation	Yes	No	Non-calcareous sedimentary rock
Currowong Granodiorite	No	No	Other rock type
Daisy Plains Leucomonzogranite	No	No	Other rock type
Dalwood Group	Yes	No	Non-calcareous sedimentary rock
Dandahra Creek Leucogranite	No	No	Other rock type
Darts Creek Mudstone	Yes	No	Non-calcareous sedimentary rock
Digby Formation, Napperby Formation	Yes	No	Non-calcareous sedimentary rock
Doctor George Granite	No	No	Other rock type
Dorrigo Mountain Complex	No	No	Other rock type
Drake Volcanics	No	No	Other rock type
Dundee Rhyodacite	No	No	Other rock type
Dundurrabin Granodiorite	No	No	Other rock type
Dunes 38496	No	No	Other rock type
Dyamberin beds	Yes	No	Non-calcareous sedimentary rock
Ebor Volcanic Complex	No	No	Other rock type
Ellenborough Volcanics	No	No	Other rock type
Ellenden Granite	No	No	Other rock type

Rock unit	Sedimentary	Calcareous	Rock type
Emmaville Volcanics	No	No	Other rock type
Emu Creek Formation	Yes	No	Non-calcareous sedimentary rock
Enmore Monzogranite	No	No	Other rock type
Estuarine and delta deposits 38489	No	No	Other rock type
Evans Head Coal Measures	Yes	No	Non-calcareous sedimentary rock
Farley Formation	Yes	No	Non-calcareous sedimentary rock
Felsic and mafic volcanics 40153	No	No	Other rock type
Felsic intrusives 42022	No	No	Other rock type
Felsic intrusives 42187	No	No	Other rock type
Felsic to intermediate intrusives 39497	No	No	Other rock type
Felsic volcanics and high level intrusives 42193	No	No	Other rock type
Fiery Range Porphyry	No	No	Other rock type
Flagstaff Formation	Yes	Yes	Calcareous sedimentary rock
Folly Volcanics	No	No	Other rock type
Gerringong Volcanics	No	No	Other rock type
Gibraltar Ignimbrite	No	No	Other rock type
Gilgai Granite	No	No	Other rock type
Gilgurry Mudstone	Yes	Yes	Calcareous sedimentary rock
Gilmore Volcanic Group	Yes	No	Non-calcareous sedimentary rock
Giro beds	Yes	No	Non-calcareous sedimentary rock
Girrakool beds, Agnes Greywacke	Yes	No	Non-calcareous sedimentary rock
Glen Esk Monzogranite	No	No	Other rock type
Glen Garry Microleucogranite	No	No	Other rock type
Glenbog Granodiorite	No	No	Other rock type
Glenclair Monzogranite	No	No	Other rock type
Glenifer Monzogranite	No	No	Other rock type
Glenrock Granodiorite	No	No	Other rock type
Glory Vale Conglomerate	Yes	No	Non-calcareous sedimentary rock
Gordonbrook Serpentinite	No	No	Other rock type
Grafton Formation	Yes	No	Non-calcareous sedimentary rock
Greta Coal Measures	Yes	No	Non-calcareous sedimentary rock
Gundahl Complex	Yes	Yes	Calcareous sedimentary rock
Gundle Granite	No	No	Other rock type
Gunning Granite	No	No	Other rock type
Hat Head Member	Yes	No	Non-calcareous sedimentary rock

Rock unit	Sedimentary	Calcareous	Rock type
Hawkesbury Sandstone	Yes	No	Non-calcareous sedimentary rock
Hawkins Volcanics	No	No	Other rock type
Henry River Granite	No	No	Other rock type
Hudsons Peak Ignimbrite Member, Johns Hill Ignimbrite Member, Martins Creek Ignimbrite Member	No	No	Other rock type
Hyndmans Creek Formation	Yes	No	Non-calcareous sedimentary rock
Illawambra Monzogranite	No	No	Other rock type
Illawarra Coal Measures	Yes	No	Non-calcareous sedimentary rock
intermediate to felsic intrusives 42179	No	No	Other rock type
Isismurra Formation	Yes	No	Non-calcareous sedimentary rock
Jeremadra Granodiorite	No	No	Other rock type
Jillicambra Monzogranite	No	No	Other rock type
Jinden Monzogranite	No	No	Other rock type
Jingo Creek Monzogranite	No	No	Other rock type
Johnsons Creek Conglomerate, McInnes Formation	Yes	No	Non-calcareous sedimentary rock
Jolly Nose Conglomerate	Yes	No	Non-calcareous sedimentary rock
Kaloe Tonalite	No	No	Other rock type
Kameruka Granodiorite	No	No	Other rock type
Kanangra Granite	No	No	Other rock type
Kangaroo Creek Sandstone	Yes	No	Non-calcareous sedimentary rock
Kangaroo Mountain Basanite	No	No	Other rock type
Karikeree Metadolerite	No	No	Other rock type
Kellys Creek Leucomonzogranite	No	No	Other rock type
Kempsey beds	Yes	No	Non-calcareous sedimentary rock
Kilburnie Monzogranite	No	No	Other rock type
Kindee Conglomerate	Yes	No	Non-calcareous sedimentary rock
Kingsgate Leucogranite	No	No	Other rock type
Kiwarrak beds	Yes	No	Non-calcareous sedimentary rock
Kookabookra Monzogranite	No	No	Other rock type
Koolanock Sandstone	Yes	No	Non-calcareous sedimentary rock
Koorainghat beds	Yes	No	Non-calcareous sedimentary rock
Kullatine Formation	Yes	No	Non-calcareous sedimentary rock
Lambie Group	Yes	No	Non-calcareous sedimentary rock
Lamington Volcanics	No	No	Other rock type

Rock unit	Sedimentary	Calcareous	Rock type
Laurieton Conglomerate	Yes	No	Non-calcareous sedimentary rock
Lett Granite	No	No	Other rock type
Liverpool Range Volcanics	Yes	No	Non-calcareous sedimentary rock
Lochaber Greywacke	Yes	No	Non-calcareous sedimentary rock
Long Flat Volcanics	No	No	Other rock type
Lords Granite	No	No	Other rock type
Louie pluton	No	No	Other rock type
Lumley Granite	No	No	Other rock type
Mackenzie Monzogranite	No	No	Other rock type
Macleay Group	Yes	Yes	Calcareous sedimentary rock
Mafic intrusives 39501	No	No	Other rock type
Mafic intrusives 42172	No	No	Other rock type
Mafic intrusives 42178	No	No	Other rock type
Mafic intrusives 42185	No	No	Other rock type
Mafic volcanic rocks 38495	No	No	Other rock type
Mafic volcanics 42177	No	No	Other rock type
Main Range Volcanics	No	No	Other rock type
Majors Creek Formation	Yes	No	Non-calcareous sedimentary rock
Mammy Johnsons Formation	Yes	No	Non-calcareous sedimentary rock
Manning Group	Yes	Yes	Calcareous sedimentary rock
Marburg Subgroup	Yes	No	Non-calcareous sedimentary rock
Marulan Granite	No	No	Other rock type
McGraths Hump Metabasalt	No	No	Other rock type
melange 69459	Yes	Yes	Calcareous sedimentary rock
Melange 75701	No	No	Other rock type
Meringo Creek Formation	Yes	No	Non-calcareous sedimentary rock
Merlewood Formation	Yes	No	Non-calcareous sedimentary rock
Merrimbula Group	Yes	No	Non-calcareous sedimentary rock
Mile Road beds	Yes	Yes	Calcareous sedimentary rock
Milton Monzonite	No	No	Other rock type
Mingaletta Formation	Yes	No	Non-calcareous sedimentary rock
Mirannie Volcanic Member	No	No	Other rock type
Mogendoura Granodiorite	No	No	Other rock type
Mole Granite	No	No	Other rock type

Rock unit	Sedimentary	Calcareous	Rock type
Moombil Siltstone	Yes	No	Non-calcareous sedimentary rock
Mooraback beds	Yes	No	Non-calcareous sedimentary rock
Morgans Creek Monzogranite	No	No	Other rock type
Moruya Tonalite	No	No	Other rock type
Mount Barney beds	Yes	Yes	Calcareous sedimentary rock
Mount Dromedary Igneous Complex	No	No	Other rock type
Mount Duval Monzogranite	No	No	Other rock type
Mount Fairy Group	Yes	Yes	Calcareous sedimentary rock
Mount Johnstone Formation	Yes	No	Non-calcareous sedimentary rock
Mount Jonblee Leucomonzogranite	No	No	Other rock type
Mount Mitchell Monzogranite	No	No	Other rock type
Mount Poole Monzogranite	No	No	Other rock type
Mount Warning Central Complex	No	No	Other rock type
Muirs Creek Conglomerate	Yes	No	Non-calcareous sedimentary rock
Mulbring Siltstone	Yes	No	Non-calcareous sedimentary rock
Mumbulla Granite	No	No	Other rock type
Muree Sandstone	Yes	No	Non-calcareous sedimentary rock
Murrabrine Quartz Diorite	No	No	Other rock type
Myra beds	Yes	No	Non-calcareous sedimentary rock
Nagha Granite	No	No	Other rock type
Nambucca beds	Yes	No	Non-calcareous sedimentary rock
Narooma Chert	Yes	No	Non-calcareous sedimentary rock
Narrabeen Group	Yes	No	Non-calcareous sedimentary rock
Nelligen Granodiorite	No	No	Other rock type
Neranleigh-Fernvale beds	Yes	No	Non-calcareous sedimentary rock
Nerong Volcanics, Berrico Creek Formation, Conger Formation	Yes	Yes	Calcareous sedimentary rock
Nevann Siltstone	Yes	No	Non-calcareous sedimentary rock
Newcastle Coal Measures	Yes	No	Non-calcareous sedimentary rock
Newtown Formation	Yes	No	Non-calcareous sedimentary rock
Nimmitabel Monzogranite	No	No	Other rock type
Nowra Sandstone	Yes	No	Non-calcareous sedimentary rock

Rock unit	Sedimentary	Calcareous	Rock type
Nungatta Granodiorite	No	No	Other rock type
Nymboida Coal Measures	Yes	No	Non-calcareous sedimentary rock
Oban River Leucomonzogranite	No	No	Other rock type
Oxley Metamorphics	Yes	No	Non-calcareous sedimentary rock
Palerang Formation	Yes	No	Non-calcareous sedimentary rock
Pappinbarra Formation	Yes	Yes	Calcareous sedimentary rock
Parkesbourne Granite	No	No	Other rock type
Parlour Mountain Leucomonzogranite	No	No	Other rock type
Parrabel beds	Yes	Yes	Calcareous sedimentary rock
Parry Group	Yes	Yes	Calcareous sedimentary rock
Paterson Volcanics	No	No	Other rock type
Pee Dee beds	Yes	No	Non-calcareous sedimentary rock
Pericoe Monzogranite	No	No	Other rock type
Pheasant Creek Volcanics	No	No	Other rock type
Pheasants Nest Formation	Yes	No	Non-calcareous sedimentary rock
Pi Pi Ignimbrite	No	No	Other rock type
Pilliga Sandstone	Yes	No	Non-calcareous sedimentary rock
Pleasant Hill Granite	No	No	Other rock type
Pollwombra Granodiorite	No	No	Other rock type
Pringles Monzogranite	No	No	Other rock type
Quaama Granodiorite	No	No	Other rock type
Razorback Creek Mudstone	Yes	Yes	Calcareous sedimentary rock
Red Cliff Coal Measures	Yes	No	Non-calcareous sedimentary rock
Red Range Microleucogranite	No	No	Other rock type
Redfern Granite	No	No	Other rock type
Ripley Road Sandstone	Yes	No	Non-calcareous sedimentary rock
Robertson Basalt	No	No	Other rock type
Rockton Granodiorite	No	No	Other rock type
Rollans Road Formation	Yes	No	Non-calcareous sedimentary rock
Rolling Downs Group	Yes	Yes	Calcareous sedimentary rock
Round Mountain Leucomonzogranite	No	No	Other rock type
Ruby Creek Granite	No	No	Other rock type
Rutherford Formation	Yes	No	Non-calcareous sedimentary rock

Rock unit	Sedimentary	Calcareous	Rock type
Ryrie Formation	Yes	No	Non-calcareous sedimentary rock
Saltwater Creek Formation	Yes	No	Non-calcareous sedimentary rock
Sand plain 38499	No	No	Other rock type
Sandon beds, Lochaber Greywacke, Oxley Metamorphics, Wybeena Metamorphics	Yes	No	Non-calcareous sedimentary rock
Sara beds	Yes	No	Non-calcareous sedimentary rock
Seaham Formation	Yes	No	Non-calcareous sedimentary rock
sedimentary rocks 72357	Yes	Yes	Calcareous sedimentary rock
sediments 39454	Yes	No	Non-calcareous sedimentary rock
sediments 39463	Yes	Yes	Calcareous sedimentary rock
sediments 39484	Yes	No	Non-calcareous sedimentary rock
Serpentinised mafic and ultramafic rocks 42190	No	No	Other rock type
Shoalhaven Group	Yes	No	Non-calcareous sedimentary rock
Silver Gully Formation	Yes	No	Non-calcareous sedimentary rock
Singleton Supergroup	Yes	No	Non-calcareous sedimentary rock
Smokey Cape Monzogranite	No	No	Other rock type
Snapper Point Formation, Wandrawandian Siltstone	Yes	No	Non-calcareous sedimentary rock
Stanthorpe Granite 1	No	No	Other rock type
Stanthorpe Granite 2	No	No	Other rock type
Stanthorpe Granite 5	No	No	Other rock type
Tallong Conglomerate	Yes	No	Non-calcareous sedimentary rock
Taralga Group	Yes	No	Non-calcareous sedimentary rock
Telegraph Point Member	Yes	No	Non-calcareous sedimentary rock
Texas beds	Yes	Yes	Calcareous sedimentary rock
The Brothers Granitoids	No	No	Other rock type
Thrumster Slate	Yes	No	Non-calcareous sedimentary rock
Tomago Coal Measures	Yes	No	Non-calcareous sedimentary rock
Tombong Formation	Yes	No	Non-calcareous sedimentary rock
Touchwood Formation	Yes	No	Non-calcareous sedimentary rock

Rock unit	Sedimentary	Calcareous	Rock type
Towamba Granodiorite	No	No	Other rock type
Trachyte 39500	No	No	Other rock type
Twofold Bay Formation	Yes	No	Non-calcareous sedimentary rock
Unnamed granitoids 42188	No	No	Other rock type
Valla Monzogranite	No	No	Other rock type
Vane Subgroup, Archerfield Sandstone	Yes	No	Non-calcareous sedimentary rock
Wadbilliga Monzogranite	No	No	Other rock type
Wagonga Group	Yes	No	Non-calcareous sedimentary rock
Walibree Formation	Yes	No	Non-calcareous sedimentary rock
Wallagaraugh Monzogranite	No	No	Other rock type
Wallanbah Formation	Yes	Yes	Calcareous sedimentary rock
Wallangarra Volcanics	No	No	Other rock type
Wallaringa Formation	Yes	No	Non-calcareous sedimentary rock
Walloon Coal Measures	Yes	No	Non-calcareous sedimentary rock
Wang Wauk Formation	Yes	No	Non-calcareous sedimentary rock
Warbro Formation	Yes	No	Non-calcareous sedimentary rock
Wards Creek beds	Yes	No	Non-calcareous sedimentary rock
Wards Mistake Monzogranite	No	No	Other rock type
Wards River Conglomerate	Yes	No	Non-calcareous sedimentary rock
Watonga Formation	Yes	No	Non-calcareous sedimentary rock
Waverley Formation	Yes	Yes	Calcareous sedimentary rock
Werrikimbe Volcanics	No	No	Other rock type
Whipstick Monzogranite	No	No	Other rock type
Whitlow Formation	Yes	No	Non-calcareous sedimentary rock
Why Worry Tonalite	No	No	Other rock type
Wianamatta Group	Yes	No	Non-calcareous sedimentary rock
Willowie Creek beds	Yes	Yes	Calcareous sedimentary rock
Wittingham Coal Measures	Yes	No	Non-calcareous sedimentary rock
Woodenbong beds	Yes	No	Non-calcareous sedimentary rock
Woogaroo Subgroup	Yes	No	Non-calcareous sedimentary rock

Rock unit	Sedimentary	Calcareous	Rock type
Woolooma Formation	Yes	No	Non-calcareous sedimentary rock
Wootton beds	Yes	Yes	Calcareous sedimentary rock
Worange Point Formation	Yes	No	Non-calcareous sedimentary rock
Wroxham Granodiorite	No	No	Other rock type
Yagon Siltstone, Booti Booti Sandstone	Yes	No	Non-calcareous sedimentary rock
Yalmy Group	Yes	No	Non-calcareous sedimentary rock
Yambulla Granodiorite	No	No	Other rock type
Yarrahapinni Monzogranite	No	No	Other rock type
Yarras Complex	No	No	Other rock type
Yarrimie Formation	No	No	Other rock type
Youdales Hut Formation	Yes	Yes	Calcareous sedimentary rock
Yurammie Granodiorite	No	No	Other rock type
