# Supplementary material for: Geology controls the distribution of a seed-eating bird: Feeding-tree selection by the glossy black-cockatoo Calyptorhynchus lathami
Source: PLoS One. 2024 Aug 8;19(8):e0308323. doi: 10.1371/journal.pone.0308323 (PMC11309512; doi:10.1371/journal.pone.0308323)
Supplement: S6 Table — Logistic modelling was undertaken using the glm function in the R package stats. (PDF) [file pone.0308323.s006.pdf]

**S6 Table. Logistic model explaining the influence of predicted Food Value on the incidence of a drooping sheoak being selected as a feeding tree.**

Logistic modelling was undertaken using the glm function in R **[1].**

| **Equation** | **Incidence of tree selection ~ predicted Food Value** | | | | |
|---|---|---|---|---|---|
| **Family** | Binomial | | | | |
| **Link function** | logit | | | | |
| **Coefficients** | **Estimate** | **Std Error** | **Z** | **P** | |
| (Intercept) | -6.210 | 1.335 | -4.65 | <0.0001 | |
| cFV | 0.2240 | 0.0464 | 4.82 | <0.0001 | |
| Null deviance | 159.35 on 114 degrees of freedom | | | | |
| Residual deviance | 121.07 on 113 degrees of freedom | | | | |
| Number of Fisher Scoring iterations | 5 | | | | |
| **Analysis of Deviance Table** | | | | | |
| | **d.f.** | **Deviance** | **Residual d.f.** | **Residual deviance** | **P** |
| NULL | | | 114 | 159.35 | |
| Constructed Food Value | 1 | 38.278 | 113 | 121.07 | <0.0001 |

## Reference


1. R Core Team. R: A language and environment for statistical computing. Version 4.3.1 (2023-06-16 ucrt) -- "Beagle Scouts". Vienna, Austria: R Foundation for Statistical Computing; 2023.
