# Supplementary material for: Geology controls the distribution of a seed-eating bird: Feeding-tree selection by the glossy black-cockatoo Calyptorhynchus lathami
Source: PLoS One. 2024 Aug 8;19(8):e0308323. doi: 10.1371/journal.pone.0308323 (PMC11309512; doi:10.1371/journal.pone.0308323)
Supplement: S7 Table — (PDF) [file pone.0308323.s007.pdf]

S7 Table. Elemental composition of drooping sheoak kernels.

	Concentration					Allocation				
	**Sample size**	**Median**	**Mean**	**Standard error**	**Coefficient of variation**	**Sample size**	**Median**	**Mean**	**Standard error**	**Coefficient of variation**
**Element**	(n)	(mg kg^{-1})	(mg kg^{-1})	(mg kg^{-1})	(%)	(n)	(% sound seed)	(% sound seed)	(% sound seed)	(%)
Nitrogen	43	71,500	71,823	646	5.9	42	94.16	94.12	0.164	1.1
Phosphorus	45	14,017	13,663	379	19	45	98.94	98.69	0.114	0.8
Potassium	45	10,809	10,710	191	12	45	87.57	87.02	0.703	5.4
Sulphur	45	4,829	4,818	52	7.3	45	93.49	92.80	0.297	2.1
Magnesium	45	4,450	4,619	123	18	45	92.19	91.30	0.424	3.1
Calcium	45	2,904	3,001	113	25	45	57.43	56.12	0.800	9.6
Zinc	45	226	231.9	6.0	17	45	90.37	88.85	0.718	5.4
Manganese	45	164	160.9	14.0	58	45	86.42	85.48	0.749	5.9
Sodium	45	84	92.51	4.80	35	45	34.75	34.93	1.54	29
Copper	45	66	69.89	3.67	35	45	87.07	86.47	1.12	8.7
Iron	45	59	61.82	2.42	26	45	27.20	28.09	1.33	32
Boron	45	18	17.38	0.46	18	45	61.06	60.44	1.21	13
