# Supplementary material for: Geology controls the distribution of a seed-eating bird: Feeding-tree selection by the glossy black-cockatoo Calyptorhynchus lathami
Source: PLoS One. 2024 Aug 8;19(8):e0308323. doi: 10.1371/journal.pone.0308323 (PMC11309512; doi:10.1371/journal.pone.0308323)
Supplement: S8 Table — Numbers in bold indicate the dominant variables for each component. PCA was undertaken using the R package psych using a covariance matrix and oblimin rotation. (PDF) [file pone.0308323.s008.pdf]

S8 Table. Loadings for principal components analysis for soils from under drooping sheoak.

Numbers in bold indicate the dominant variables for each component. PCA was undertaken using the *R* package *psych* **[1]** using a covariance matrix and oblimin rotation.

**Variable**	**SALINITY**	**ACIDITY**
**Loadings**		
pH (water extraction)	0.171	**-0.906**
pH ($CaCl_2$ extraction)	0.296	**-0.888**
Aluminium	-0.265	**0.784**
Iron	0.439	**0.732**
Potassium	0.591	0.619
Exchangeable potassium	0.595	0.606
Nitrogen as ammonium	0.480	0.501
Nitrogen as nitrate	0.553	-0.251
Exchangeable calcium	0.610	-0.506
Organic carbon	0.676	0.453
Phosphorus	0.697	-0.173
Sulphur	**0.818**	-0.203
Electrical conductivity	**0.828**	-0.241
Exchangeable Na	**0.856**	-0.047
Exchangeable Mg	**0.891**	0.021
SS loadings	5.77	4.42
Proportion of variance	0.384	0.295
Cumulative variance	0.384	0.679

Reference

1. Revelle W. psych: Procedures for Personality and Psychological Research. Version 2.2.3s ed: Northwestern University, Evanston, Illinois, USA; 2022.
