# Supplementary material for: Geology controls the distribution of a seed-eating bird: Feeding-tree selection by the glossy black-cockatoo Calyptorhynchus lathami
Source: PLoS One. 2024 Aug 8;19(8):e0308323. doi: 10.1371/journal.pone.0308323 (PMC11309512; doi:10.1371/journal.pone.0308323)
Supplement: S9 Table — Vector fitting undertaken using the envfit function from the R package vegan. * Vector scores adjusted for R2. (PDF) [file pone.0308323.s009.pdf]

S9 Table. Vectors for calculated soil variables and seed variables fit to principal component analysis for soils from under drooping sheoak.

Vector fitting undertaken using the envfit function from the R package vegan [1]. * Vector scores adjusted for R^2.

Variable	SALINITY score	ACIDITY score	R^2	P	Adjusted SALINITY score*	Adjusted ACIDITY score*
**Soil vectors**						
Total nitrogen	0.972	0.235	0.389	0.0002	0.606	0.146
Ammonium: nitrate	-0.002	1.000	0.121	0.0158	-0.001	0.348
Carbon: nitrogen	-0.986	0.167	0.119	0.0084	-0.340	0.057
**Seed vectors**						
Predicted Food Value	-0.445	0.895	0.093	0.0260	-0.136	0.273
Seed Fill	-0.543	0.840	0.149	0.0020	-0.210	0.325
Kernel Ratio	0.999	-0.051	0.012	0.6157	0.108	-0.005

Reference

1. Oksanen J, Blanchet FG, Friendly M, Kindt R, Legendre P, McGlinn D, et al. vegan: Community ecology package. R package. Version 2.5-7 ed2020.
