# Supplementary material for: Geology controls the distribution of a seed-eating bird: Feeding-tree selection by the glossy black-cockatoo Calyptorhynchus lathami
Source: PLoS One. 2024 Aug 8;19(8):e0308323. doi: 10.1371/journal.pone.0308323 (PMC11309512; doi:10.1371/journal.pone.0308323)
Supplement: S10 Table — Generalized additive modelling was undertaken using the R package mgcv. (PDF) [file pone.0308323.s010.pdf]

S10 Table. Generalized additive models explaining incidence of feeding being recorded in 1-ha grid cell in relation to tree species and rock type, after accounting for spatial variation.

Generalized additive modelling was undertaken using the *R* package *mgcv* [1].

Equation	Feeding record ~ Species + Rock type + location				Feeding records ~ Species * Rock type + location			
Interaction	No				Yes			
Family	Binomial				Binomial			
Link function	Logit				Logit			
Adjusted r^2	0.0513				0.0512			
Deviance explained (%)	11.2				11.2			
**Variable**	**Estimate**	**se**	**Z**	**P**	**Estimate**	**se**	**Z**	**P**
**Intercept**	-5.577	0.146	-39.51	< 0.0001	-5.788	0.222	-26.06	< 0.0001
**Species** (Forest oak versus Black sheoak)	1.176	0.094	12.45	< 0.0001	1.200	0.231	5.20	< 0.0001
**Rock type** (Non-calcareous sedimentary versus Other)	1.027	0.106	9.67	< 0.0001	1.049	0.223	4.71	< 0.0001
**Interaction**	-	-	-	-	-0.0289	0.2512	-0.115	0.908
**Random effects (basis type = random effects)**								
	**Effective df**	**Ref. df**	X^2	**p**	**Effective df**	**Ref. df**	X^2	**P**
**Location (x, y)**	0.996	1	0.996	< 0.0001	0.996	1	0.996	< 0.0001
**Analysis of deviance test (X^2)**								
	**Residual df**		**Residual deviance**		**Residual df**		**Residual deviance**	
	17613		6508.643		17612		6508.629	
**df**	1							
**Deviance**	0.0141							
**P**	0.905							
**Akaike and Bayesian Information Criteria**								
**df**	3.999985				4.999985			
**AIC**	6516.643				6518.629			
**δAIC**	1.986							
**BIC**	6547.749				6557.512			
**δBIC**	9.763							

Reference

1. Wood SN. mgcv: Mixed GAM Computation Vehicle with Automatic Smoothness Estimation. 2019.
