# Supplementary material for: Geology controls the distribution of a seed-eating bird: Feeding-tree selection by the glossy black-cockatoo Calyptorhynchus lathami
Source: PLoS One. 2024 Aug 8;19(8):e0308323. doi: 10.1371/journal.pone.0308323 (PMC11309512; doi:10.1371/journal.pone.0308323)
Supplement: S11 Table — Bold text in feeding odds field highlights feeding odds greater than 0.10. Bold text in geological description field highlights calcareous rock components. Sedimentary rocks with minor or rare calcareous components were classified as non-calcareous. (PDF) [file pone.0308323.s011.pdf]

**S11 Table. Geological units with at least 50 grid cells containing forest oak and/or black sheoak, and with feeding odds greater than 0.10.**

*Bold text in feeding odds field highlights feeding odds greater than 0.10. ** Bold text in geological description field highlights calcareous rock components. Sedimentary rocks with minor or rare calcareous components were classified as non-calcareous.

| Geological unit | Group | Feeding | Non-feeding | Feeding odds* | Geological description ** |
|---|---|---|---|---|---|
| | | | **Non-calcareous sedimentary rocks** | | |
| Brooklana beds | Grid cells | 275 | 191 | **1.440** | Thinly bedded siliceous mudstone and siltstone with rare lithofeldspathic wacke, locally chert, jasper, magnetite-bearing chert and metabasalt. |
| | Forest oak | 258 | 153 | **1.686** | |
| | Black sheoak | 17 | 55 | **0.309** | |
| Yagon Siltstone, Booti Booti Sandstone | Grid cells | 16 | 57 | **0.281** | Fossiliferous siltstone, mudstone, sandstone, white siltstone and coal-rich, crossbedded sandstone; minor volcanic member. |
| | Forest oak | 16 | 52 | **0.308** | |
| | Black sheoak | - | 5 | - | |
| Coramba beds | Grid cells | 152 | 619 | **0.246** | Lithofeldspathic wacke, minor siltstone, siliceous siltstone, mudstone, metabasalt, chert and jasper***, rare calcareous siltstone and felsic volcanics***. |
| | Forest oak | 151 | 527 | **0.287** | |
| | Black sheoak | 1 | 92 | 0.011 | |
| Pappinbarra Formation | Grid cells | 26 | 113 | **0.230** | Turbiditic lithic and volcanolithic sandstone and interbedded siltstone with ***minor*** conglomerate, tuff, calcareous sandstone, crinoidal sandstone and ***limestone***. |
| | Forest oak | 23 | 82 | **0.280** | |
| | Black sheoak | 3 | 31 | 0.097 | |
| Camden Haven Group | Grid cells | 23 | 101 | **0.228** | Quartz and quartz-lithic sandstone and carbonaceous and micaceous siltstone. |
| | Forest oak | 22 | 78 | **0.282** | |
| | Black sheoak | 1 | 23 | 0.043 | |

| Geological unit | Group | Feeding | Non-feeding | Feeding odds* | Geological description ** |
|---|---|---|---|---|---|
| | | | **Non-calcareous sedimentary rocks** | | |
| Laurieton Conglomerate | Grid cells | 12 | 64 | **0.188** | Coarse jasper, quartz and chert bearing conglomerate, minor sandstone and siltstone to the west. Reddish to grey poorly to moderately sorted pebble to cobble conglomerate. Sandstone beds uncommon. Reddish-purple mudstones absent. |
| | Forest oak | 12 | 43 | **0.279** | |
| | Black sheoak | 1 | 21 | - | |
| Koolanock Sandstone | Grid cells | 7 | 43 | **0.163** | Brown to grey lithic sandstone, interbedded bioturbated siltstone, cream rhyolite flows, upward fining conglomerate, carbonaceous siltstone and minor coal seams. |
| | Forest oak | 6 | 32 | **0.188** | |
| | Black sheoak | 1 | 11 | 0.091 | |
| Bundamba Group | Grid cells | 12 | 111 | **0.108** | Brown to grey lithic sandstone, interbedded bioturbated siltstone, cream rhyolite flows, upward fining conglomerate, carbonaceous siltstone and minor coal seams. |
| | Forest oak | 12 | 87 | **0.138** | |
| | Black sheoak | - | 24 | - | |
| Nambucca beds | Grid cells | 51 | 480 | **0.106** | Slate, phyllite, schist, lithofeldspathic sandstone, minor conglomerate. |
| | Forest oak | 48 | 437 | **0.110** | |
| | Black sheoak | 3 | 43 | 0.070 | |
| | | | **Calcareous sedimentary rocks** | | |
| Wootton beds | Grid cells | 10 | 47 | **0.213** | Thin bedded brown - grey, barren mudstone and siltstone with interbeds of lithic sandstone (frequently turbiditic and conglomerate in lower sections; upper parts with fossiliferous grey to brown mudstone, thinner bedded lithic sandstone and ***oolitic lime***. |
| | Forest oak | 10 | 36 | **0.278** | |
| | Black sheoak | 1 | 11 | - | |
