# Supplementary figures and images for: Geology controls the distribution of a seed-eating bird: Feeding-tree selection by the glossy black-cockatoo Calyptorhynchus lathami
Source: PLoS One. 2024 Aug 8;19(8):e0308323. doi: 10.1371/journal.pone.0308323 (PMC11309512; doi:10.1371/journal.pone.0308323)

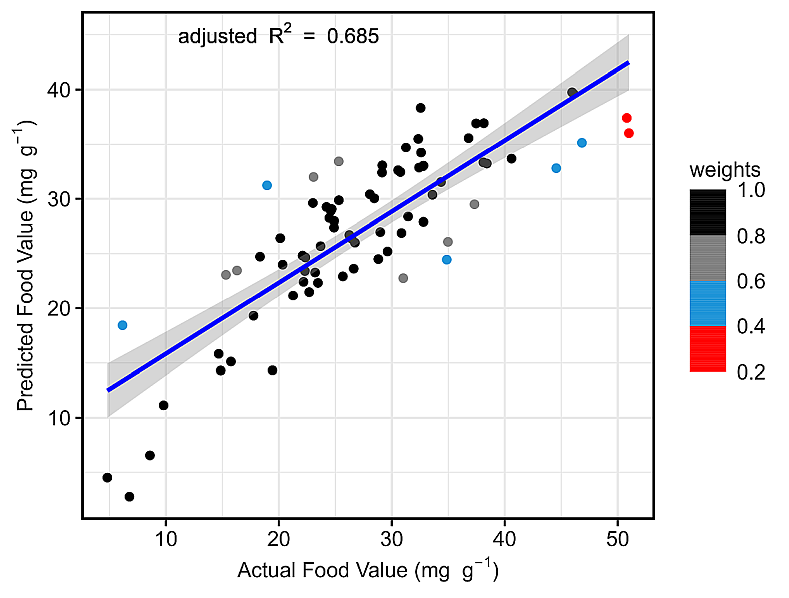

Supplement: S1 Fig — FV = Food value; SF = Seed Fill; KR = Kernel ratio. Robust regression modelling was undertaken using the lmrob function in in the R package robustbase. The influence of cases with large residuals was down-weighted based on a bi-square redescending score function. (TIF) [file pone.0308323.s012.tif]

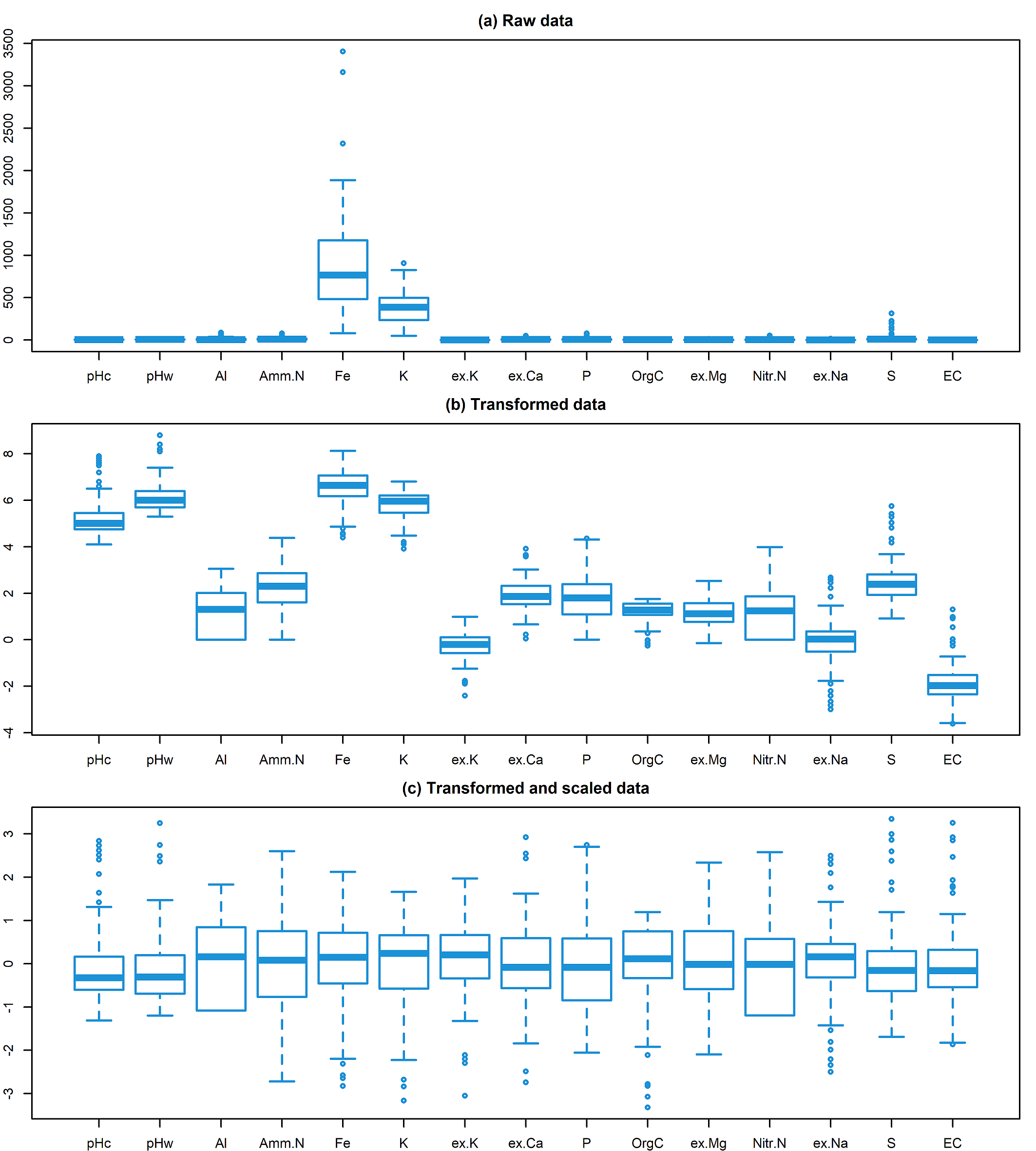

Supplement: S2 Fig — pHc and pHw were only scaled, Al was fourth-root-transformed and scaled, all other variables were log transformed and scaled. Abbreviations: pHc, pH (CaCl2 extraction); pH (water extraction); Al, aluminium; Amm.N, nitrogen as ammonium; Fe, iron; K, potassium; ex.K; exchangeable potassium; ex.Ca, exchangeable calcium; P, phosphorus; OrgC, organic carbon; ex.Mg, exchangeable magnesium; Nitr.N, N as nitrate; ex.Na, exchangeable sodium; S, sulphur; EC, electrical conductivity. Plots were drawn using boxplot in the R graphics package. (TIF) [file pone.0308323.s013.tif]

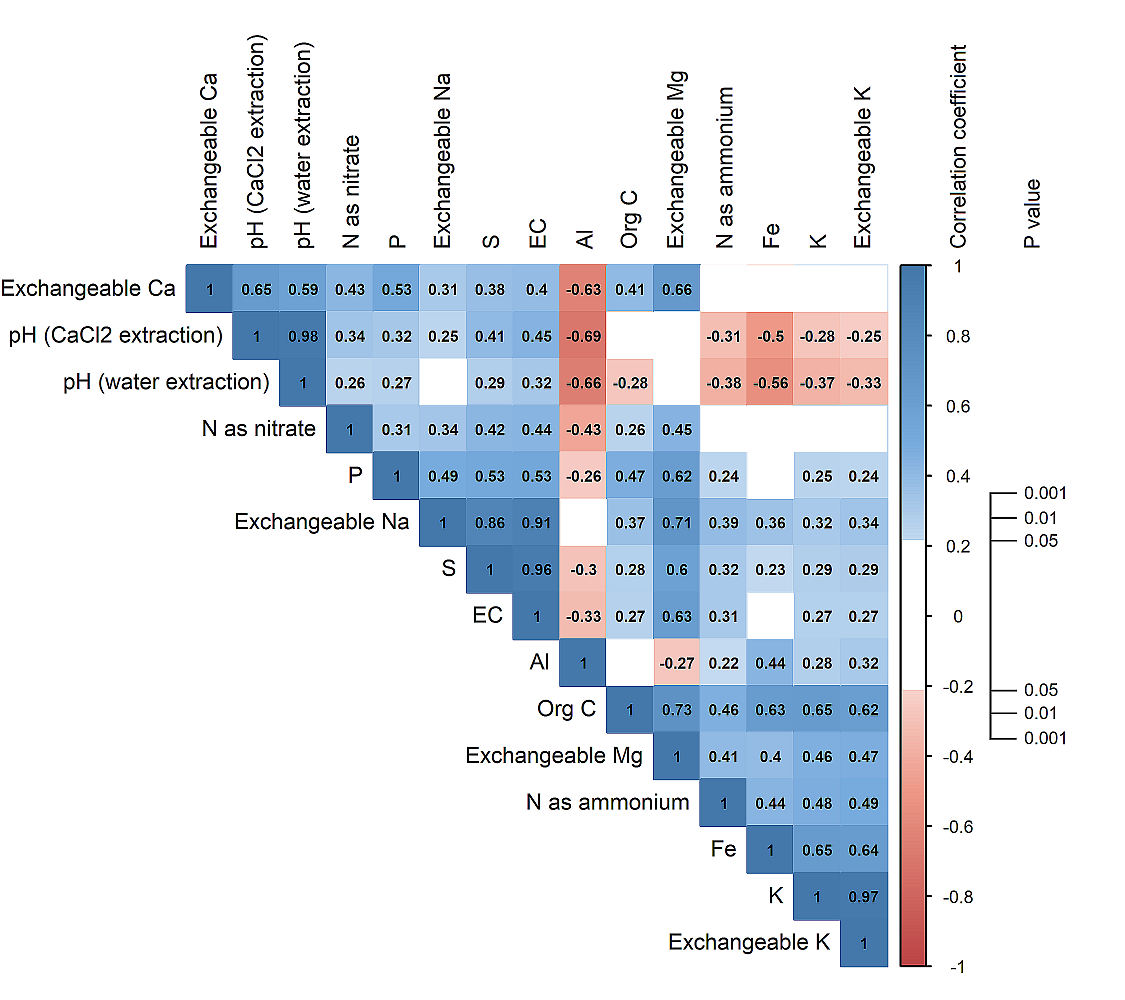

Supplement: S3 Fig — Correlation coefficients shown if P > 0.05. Correlogram was drawn using corrplot from the R package corrplot. (TIF) [file pone.0308323.s014.tif]

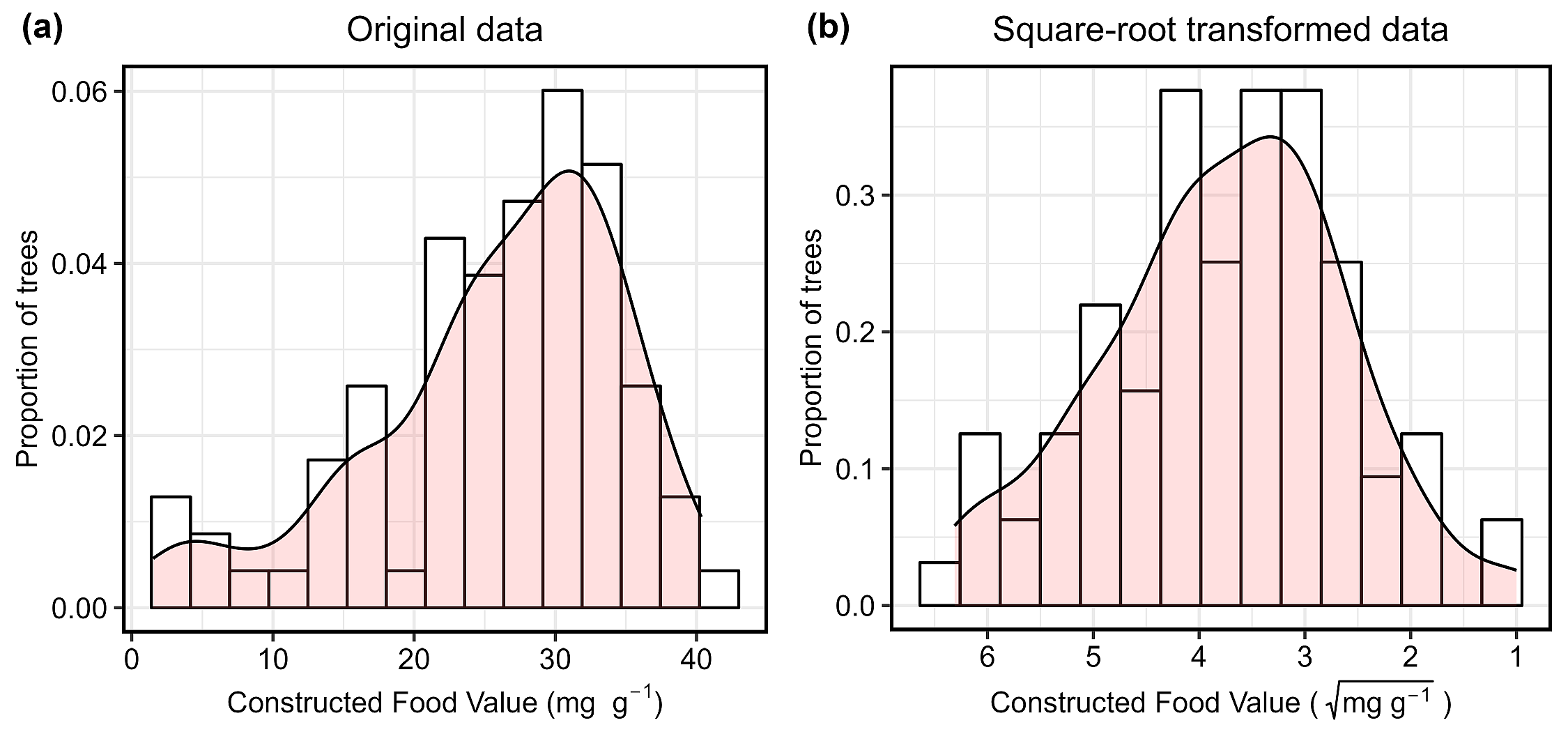

Supplement: S4 Fig — (a) original data, and (b) square-root transformed data. Plot was generated using the R package ggplot2. (TIF) [file pone.0308323.s015.tif]

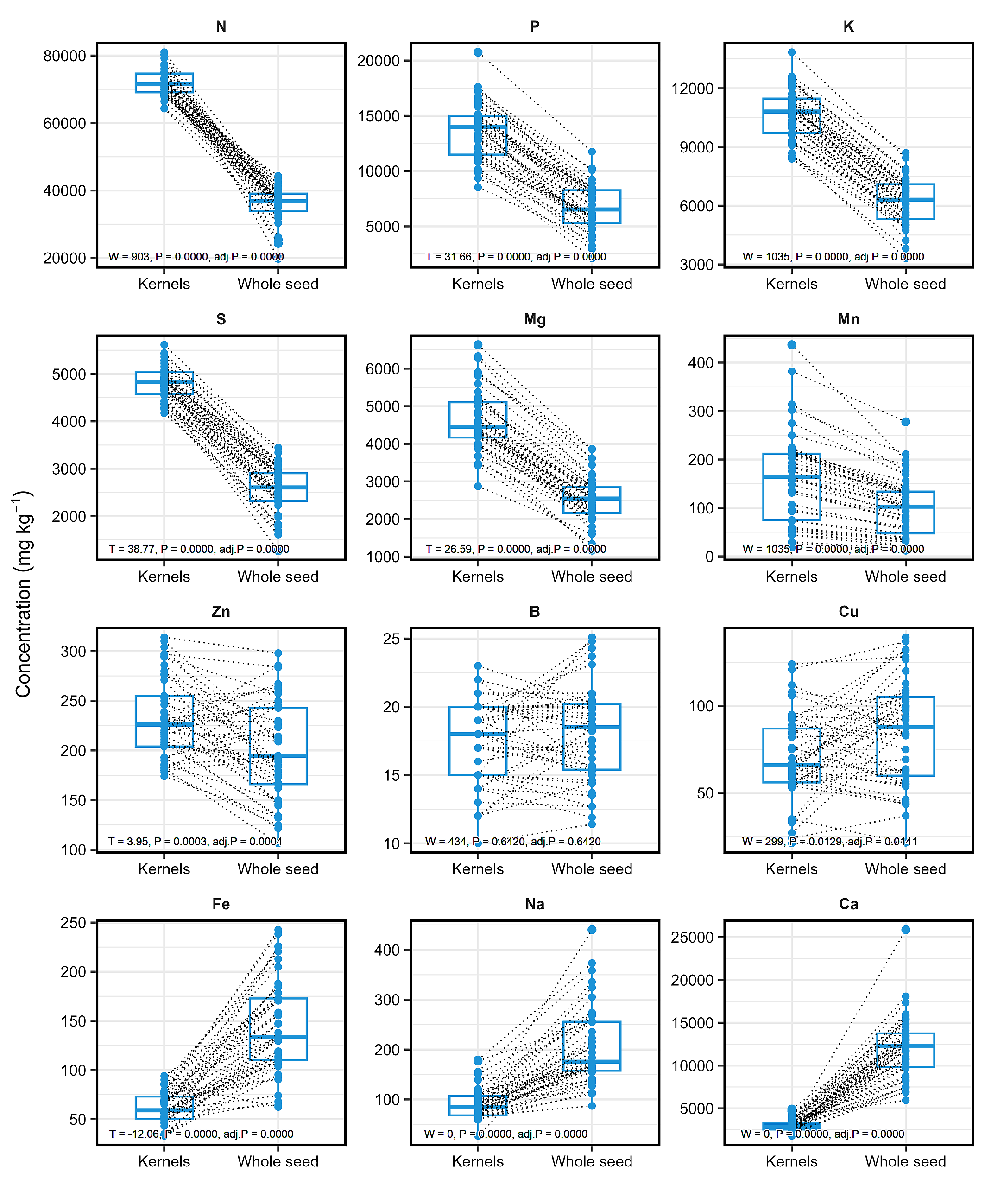

Supplement: S5 Fig — Each kernel sample was paired with the whole seed batch from which they were extracted. Sample size for nitrogen was 43, and for all other nutrients was 45. Paired t-tests were used where assumptions of normality and extreme outliers were met. Otherwise, pair-wise Wilcoxon tests were used. Plots are annotated with the relevant test statistics, P values, and the Benjamini-Hochberg-adjusted P values (adj.P). Sample sizes are in brackets. Plots were drawn using the R package ggplot2. (TIF) [file pone.0308323.s016.tif]

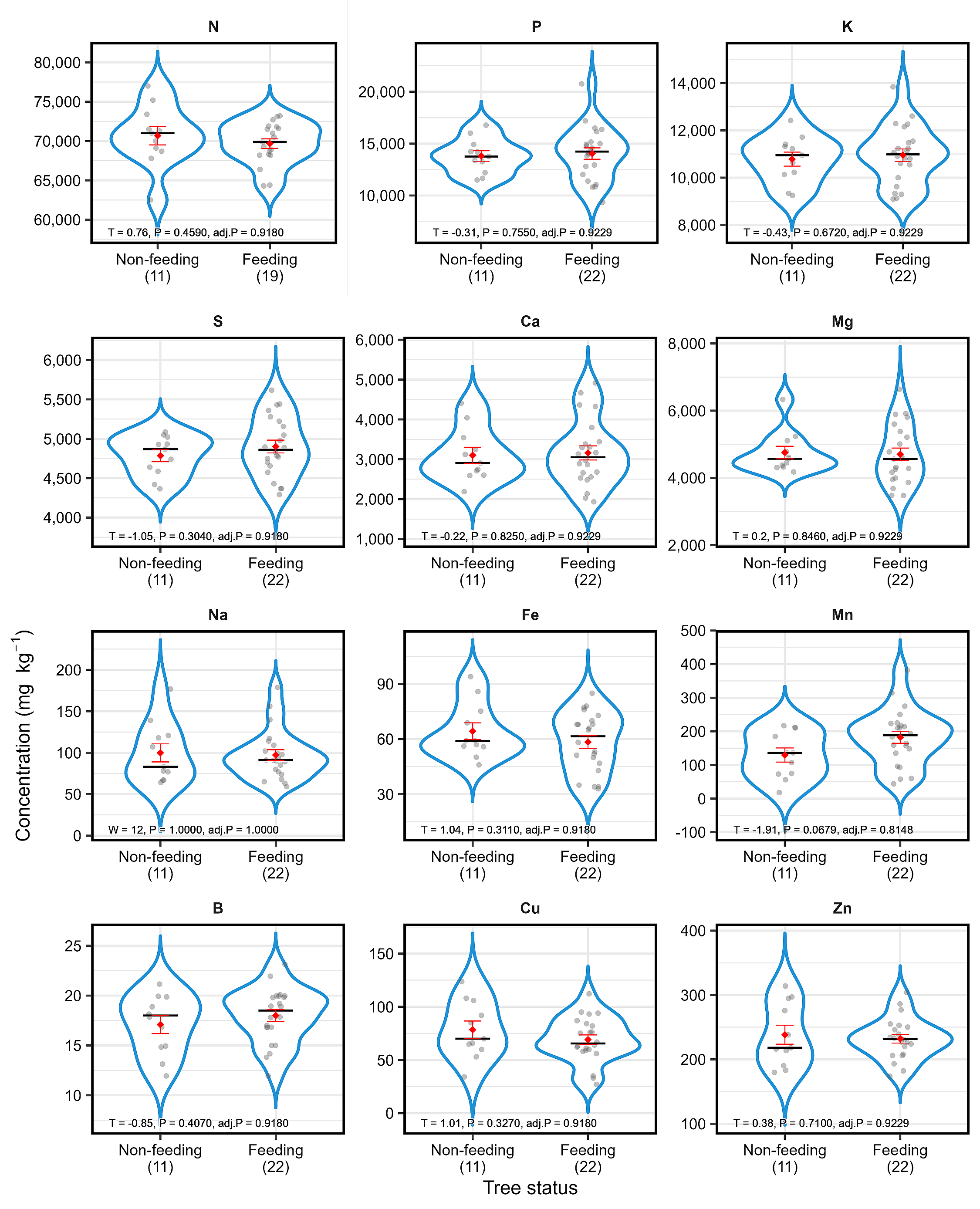

Supplement: S6 Fig — t-tests were used where assumptions of normality, equal variance and extreme outliers were met. Otherwise, Wilcoxon tests were used. Plots are annotated with the relevant test statistics, P values, and the Benjamini-Hochberg-adjusted P values (adj.P). Sample sizes are in brackets. Plots were drawn using the R package ggplot2. (TIF) [file pone.0308323.s017.tif]

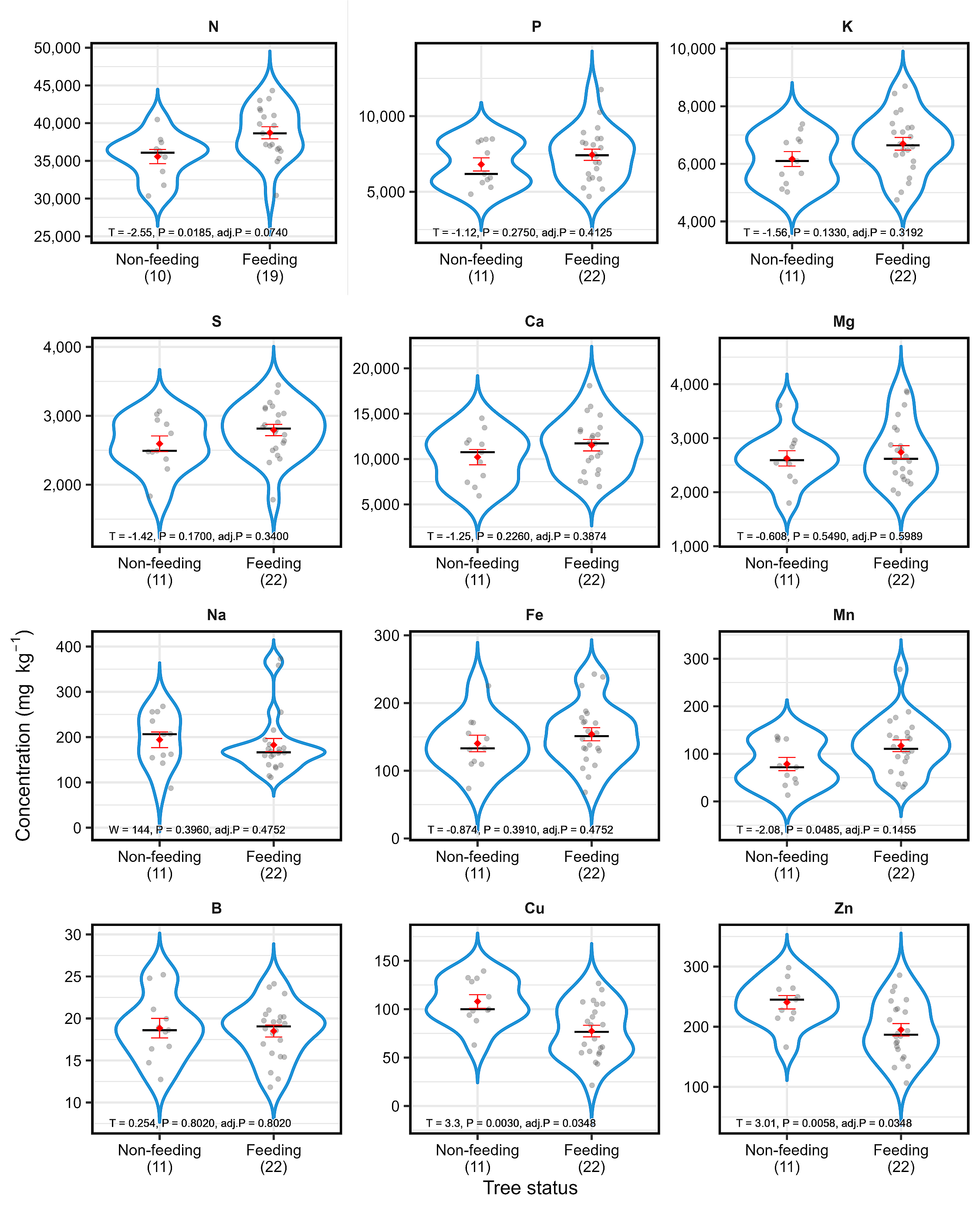

Supplement: S7 Fig — t-tests were used where assumptions of normality, equal variance and extreme outliers were met. Otherwise, Wilcoxon tests were used. Plots are annotated with the relevant test statistics, P values, and the Benjamini-Hochberg-adjusted P values (adj.P). Sample sizes are in brackets. Plots were drawn using the R package ggplot2. (TIF) [file pone.0308323.s018.tif]

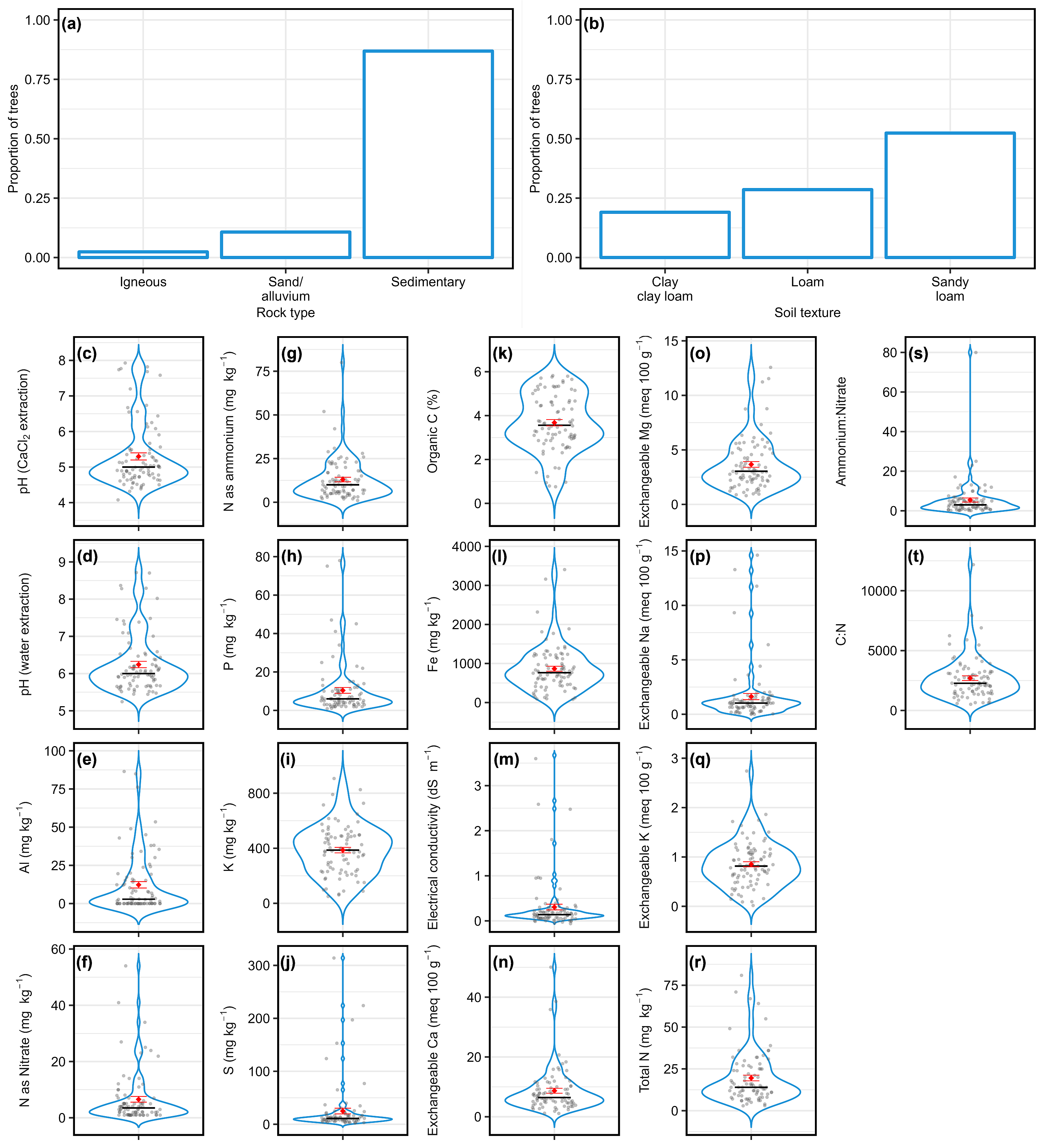

Supplement: S8 Fig — (a) rock type, (b) soil texture, (c) pH (CaCl2 extraction), (d) pH (water extraction), (e) aluminium, (f) nitrogen as nitrate, (g) nitrogen as ammonium, (h) phosphorus, (i) potassium, (j) sulphur, (k) organic carbon, (l) iron, (m) electrical conductivity, (n) exchangeable calcium, (o) exchangeable magnesium, (p) exchangeable sodium, (q) exchangeable potassium, (r) total nitrogen, (s) ammonium N:nitrate N, and (t) carbon:nitrogen. Sample was 84 size for all samples. Plots were drawn using the R package ggplot2. (TIF) [file pone.0308323.s019.tif]

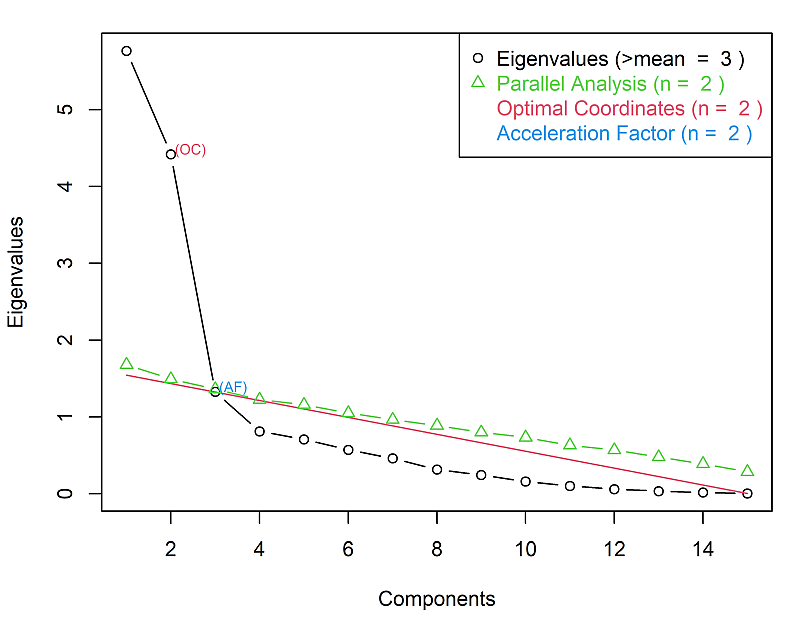

Supplement: S9 Fig — Plot was generated using nScree from the R package nFactors. (TIF) [file pone.0308323.s020.tif]

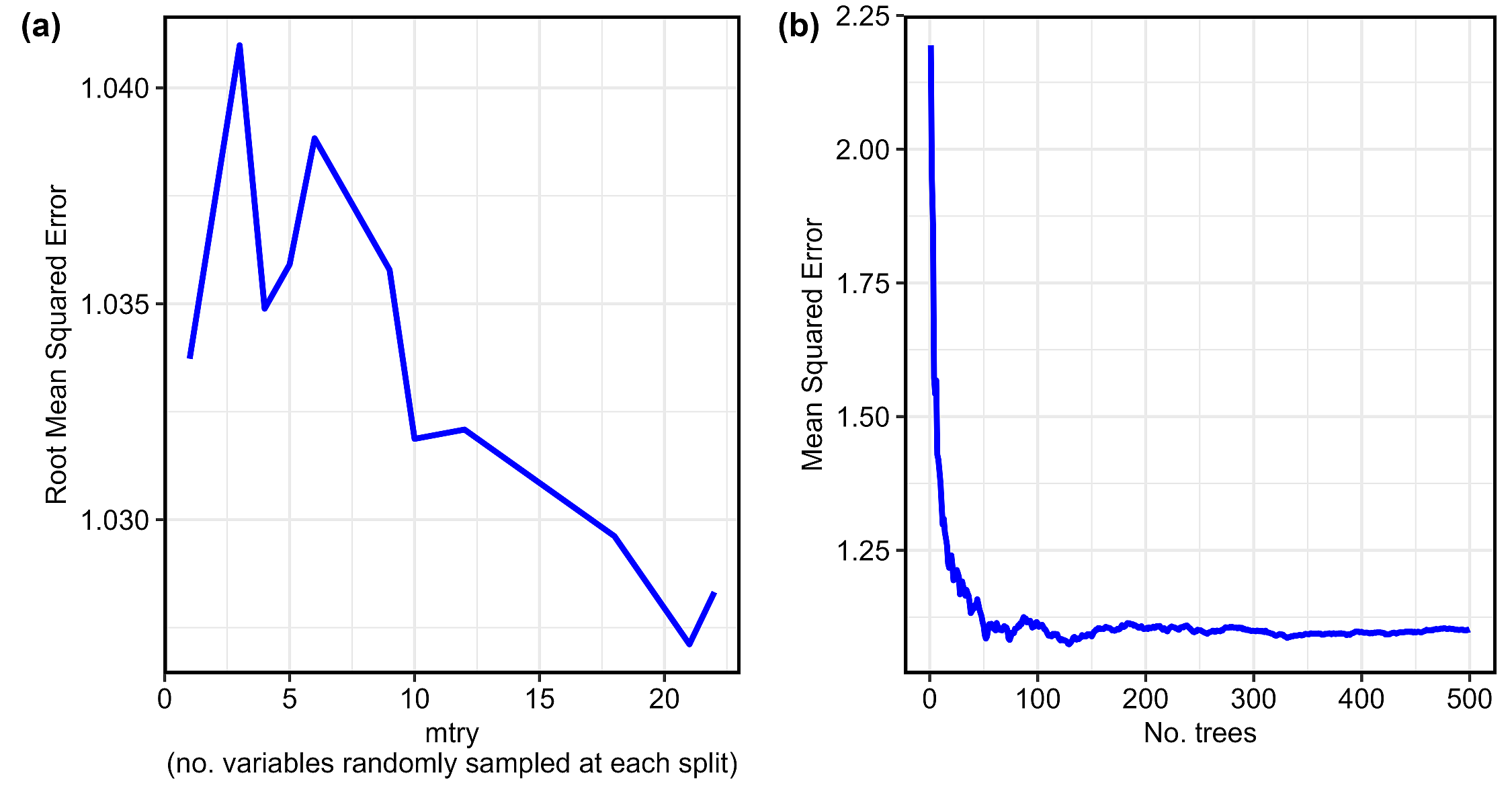

Supplement: S10 Fig — (a) Selection of the number of candidate splits to consider at each split (mtry = 21) based on lowest Root Mean Squared Error. (b) Selection of number of trees (500) based on the stabilisation of Mean Squared Error. Selection of mtry was undertaken using the train function in the R package caret. Assessment of Mean Squared Error was undertaken using the R package randomForest. Plots were generated using the R package ggplot2. (TIF) [file pone.0308323.s021.tif]

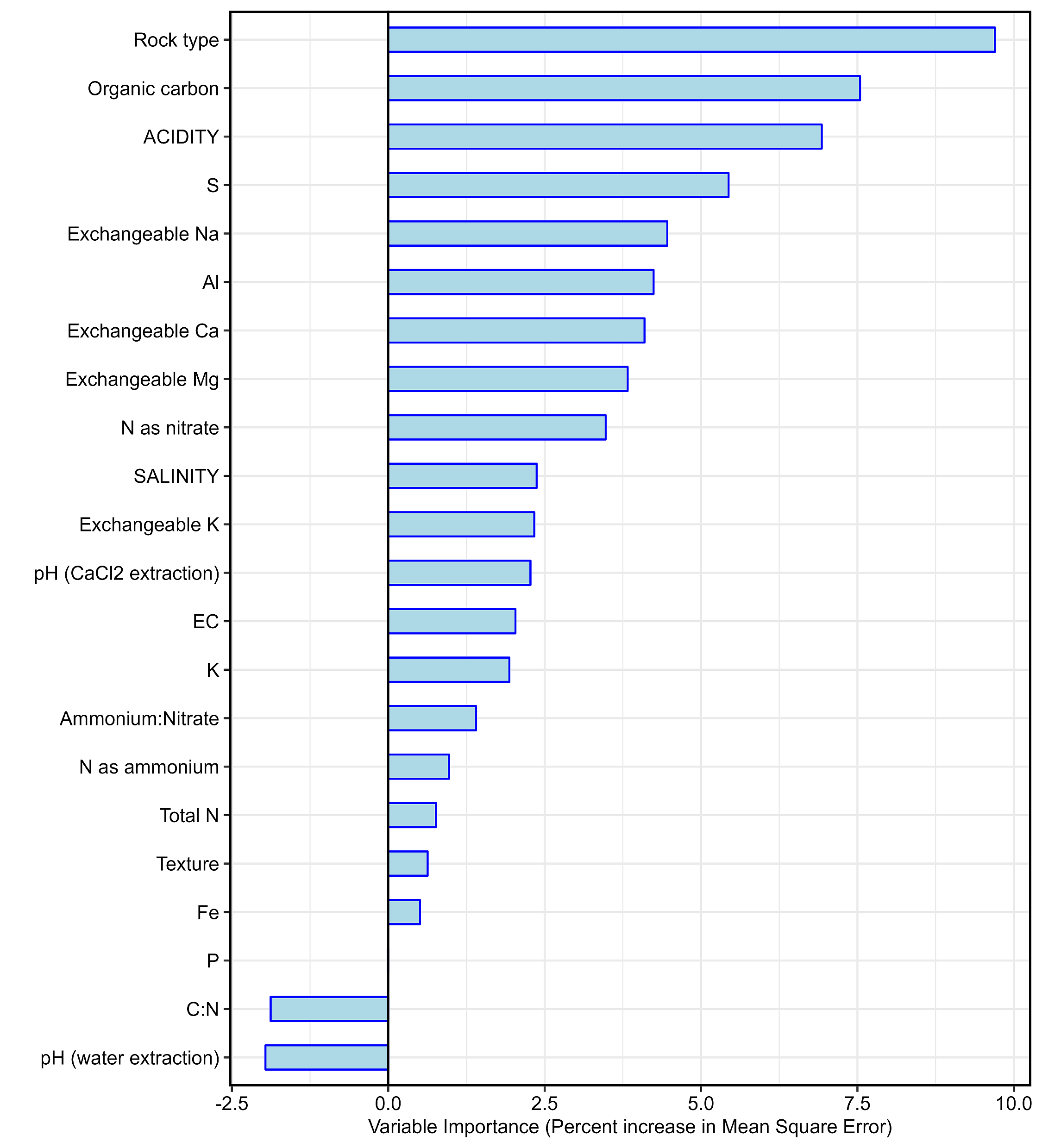

Supplement: S11 Fig — Variable Importance was calculated using R package randomForest and plotted using the R package ggplot2. (TIF) [file pone.0308323.s022.tif]

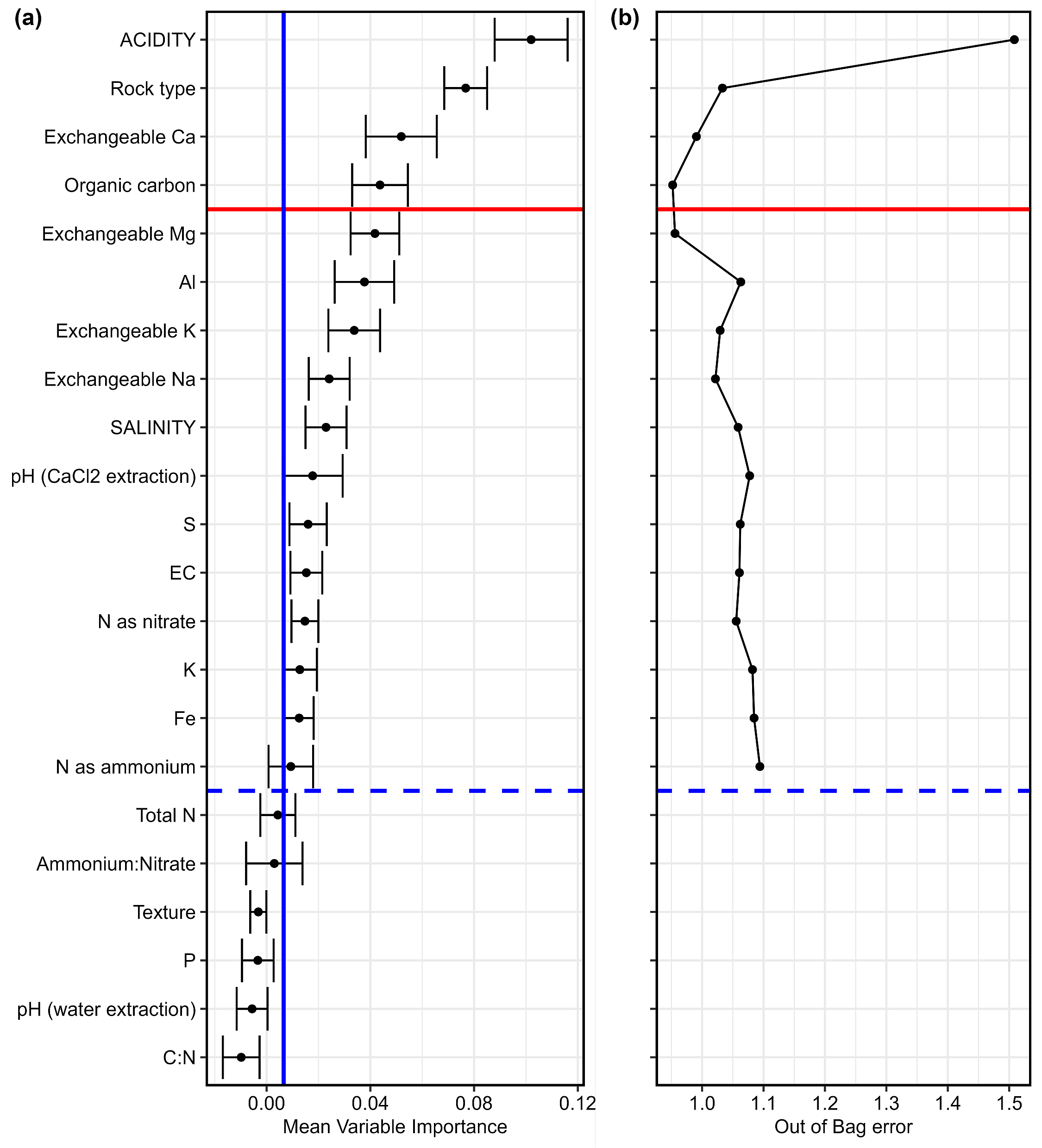

Supplement: S12 Fig — (a) Mean Variable Importance (VI) and (b) Out of Bag (OOB) error. Solid blue line is the threshold for variable inclusion in the modelling (based on VI standard deviation exceeding a minimum value predicted by a pruned classification and regression tree fitted the standard deviation curve). Dashed blue line indicates variables excluded by this selection process. Solid red line is the threshold for variable inclusion in the final model (based on a decrease in the OOB error). Variable selection was undertaken using the R package VSURF. Plots were generated using the R package ggplot2 [127]. (TIF) [file pone.0308323.s023.tif]

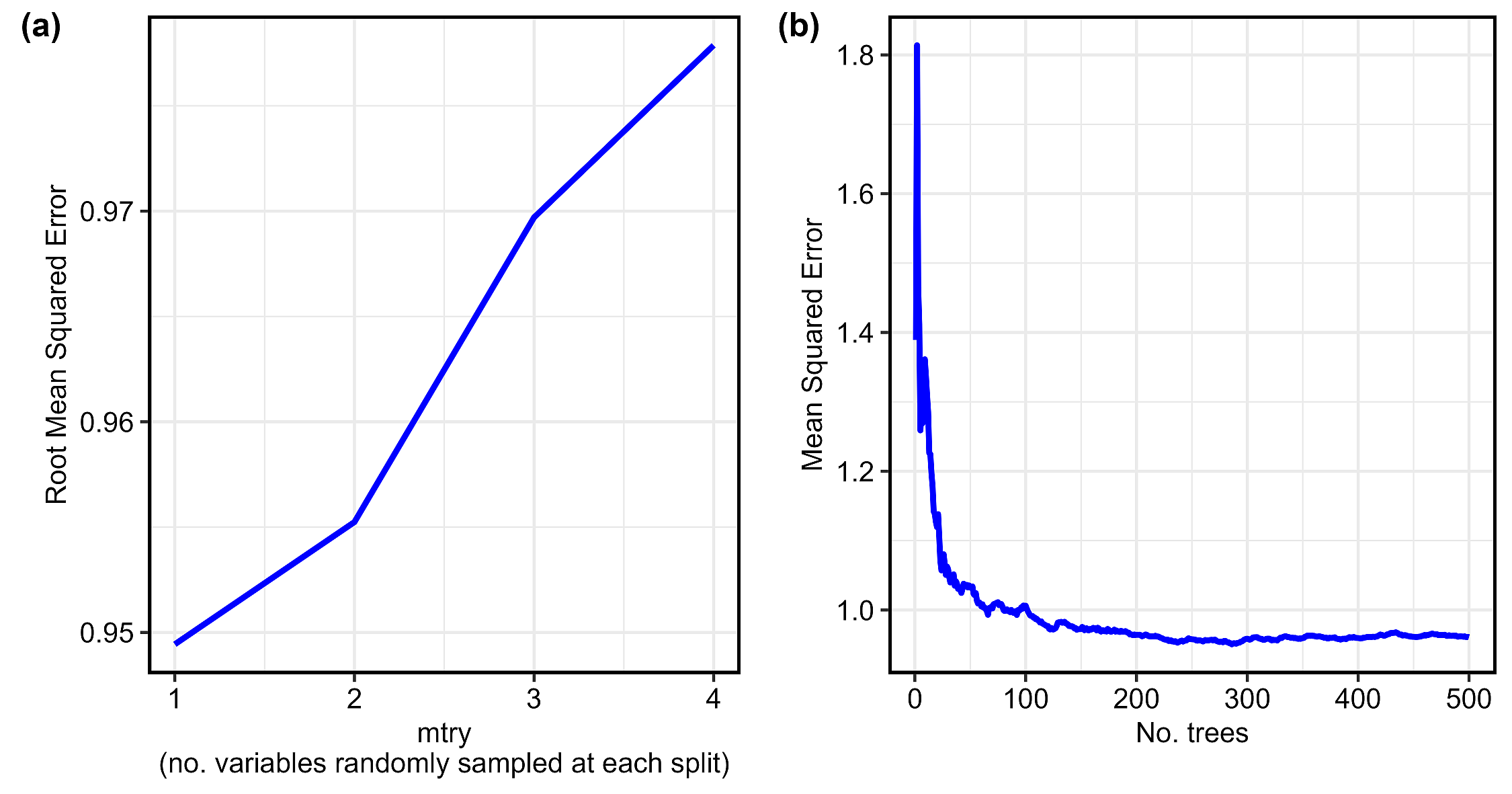

Supplement: S13 Fig — (a) Selection of the number of candidate splits to consider at each split (mtry = 1) based on lowest Root Mean Squared Error. (b) Selection of number of trees (500) based on stabilisation of Mean Squared Error. Selection of mtry was undertaken using the train function in the R package caret. Assessment of Mean Squared Error was undertaken using the R package randomForest. Plots were generated using the R package ggplot2. (TIF) [file pone.0308323.s024.tif]

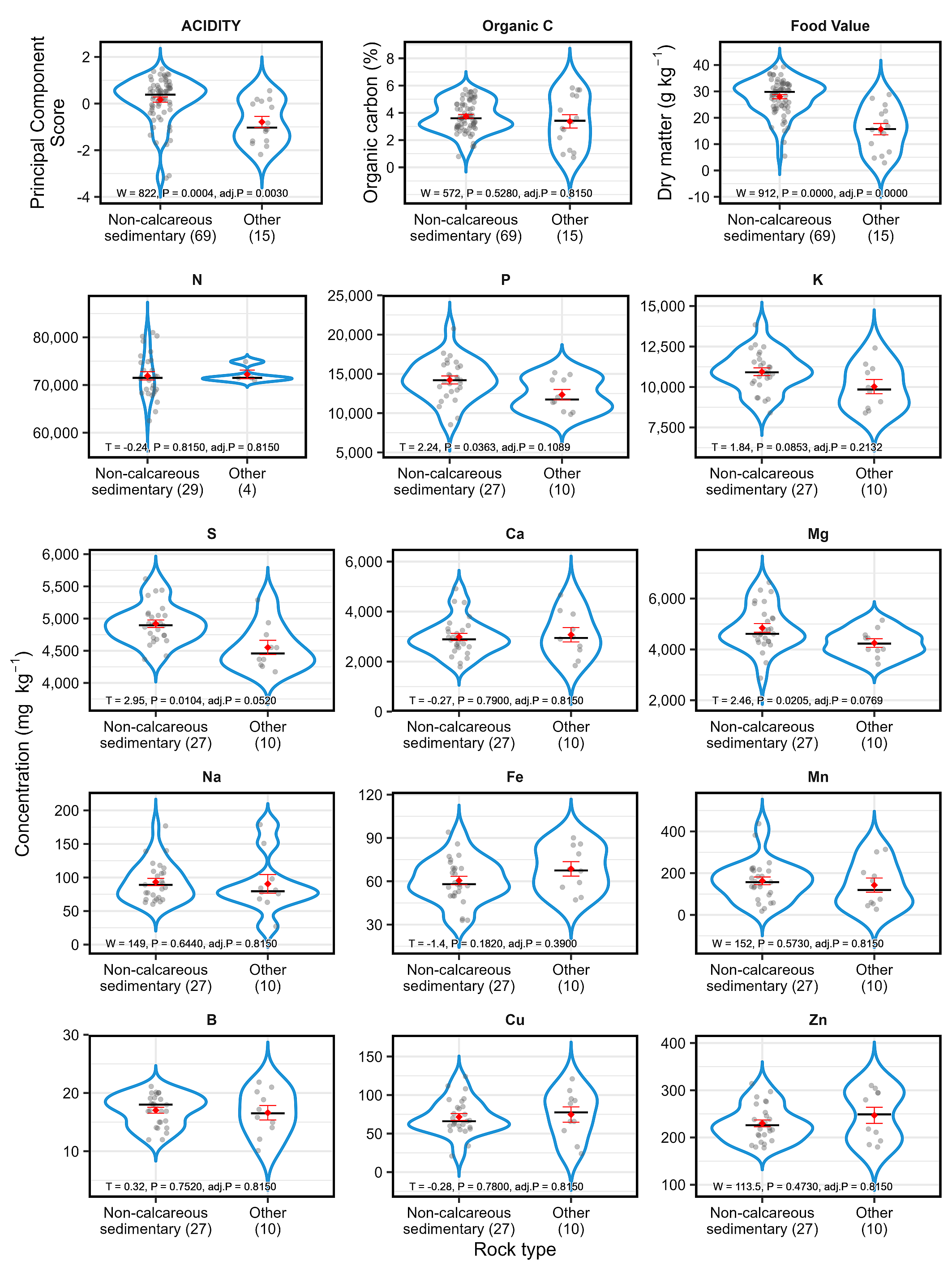

Supplement: S14 Fig — t-tests were used where assumptions of normality, equal variance and extreme outliers were met. Otherwise, Wilcoxon tests were used. Plots are annotated with the relevant test statistics, P values, and the Benjamini-Hochberg-adjusted P values (adj.P). Sample sizes are in brackets. Plots were drawn using the R package ggplot2. (TIF) [file pone.0308323.s025.tif]

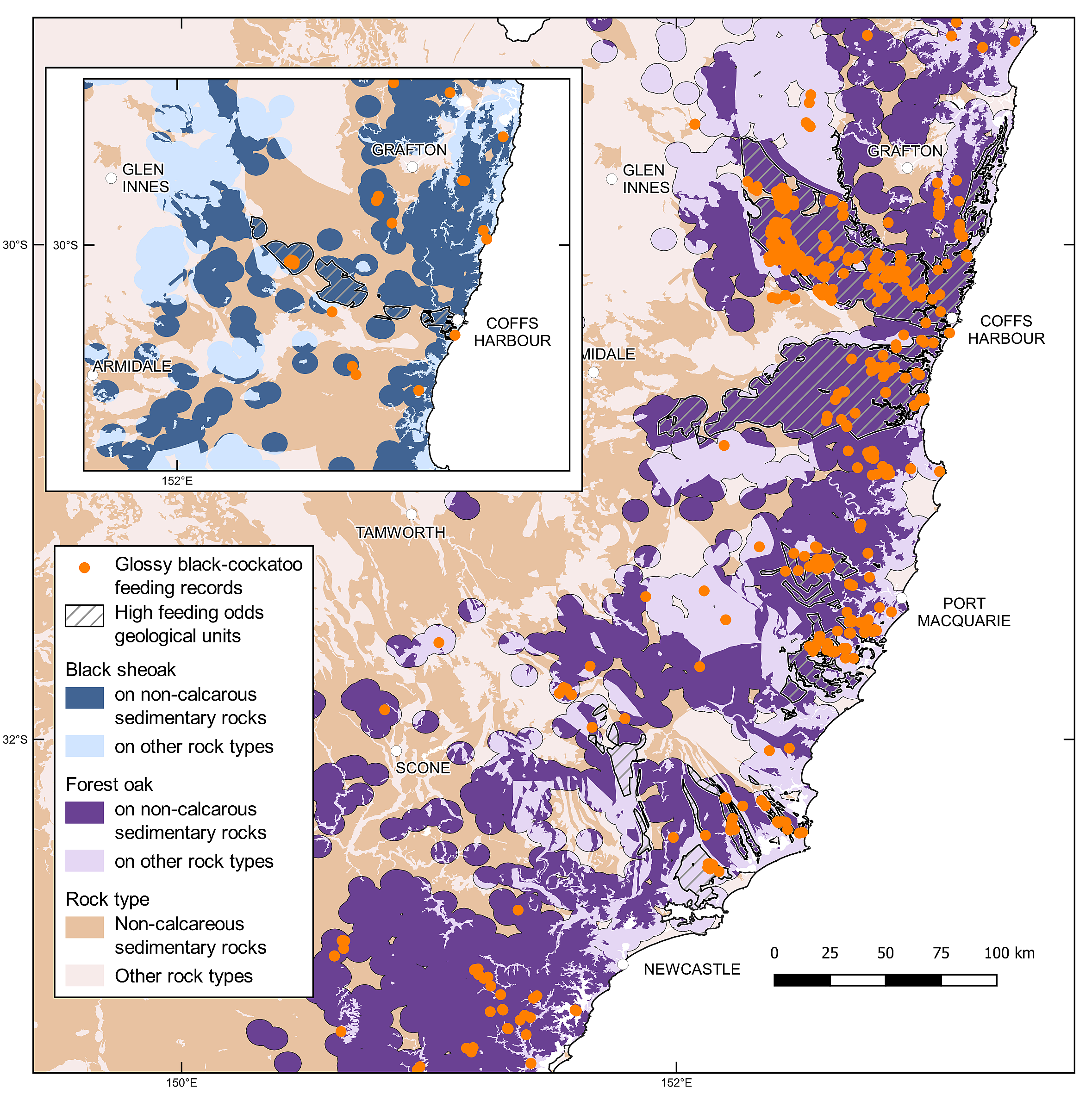

Supplement: S15 Fig — Geological units with a feeding odds of at least 0.1 for either forest sheoak or black sheoak. (TIF) [file pone.0308323.s026.tif]
