# Supplementary material for: Geology controls the distribution of a seed-eating bird: Feeding-tree selection by the glossy black-cockatoo Calyptorhynchus lathami
Source: PLoS One. 2024 Aug 8;19(8):e0308323. doi: 10.1371/journal.pone.0308323 (PMC11309512; doi:10.1371/journal.pone.0308323)
Supplement: Attachment — Submitted filename: Response to Reviewers.docx [file pone.0308323.s029.docx]

| Daniel de Paiva Silva Academic Editor PLOS ONE  10 June 2024 |  | Gabriel Crowley University of Adelaide [gabriel.m.crowley@gmail.com](mailto:gabriel.m.crowley@gmail.com) |
| --- | --- | --- |

**Response to reviewers of
Geology controls the distribution of a seed-eating bird: Feeding-tree selection by the glossy black-cockatoo *Calyptorhynchus lathami***

Reviewer #1: The scientific manuscript entitled “Geology controls the distribution of a seed-eating bird: Feeding-tree selection by the glossy black-cockatoo Calyptorhynchus lathami”, is interesting for addressing geological aspects that shape the presence of casuarinas and therefore the distribution of the birds. The introductory part is very well written, however it is specifically missing to describe other variables that also limit the distribution of birds, as well as to describe the process of co-evolution or adaptation of the bird in depending strictly on that food resource. The problem statement must be highlighted in a better way, that is, the problem behind it and therefore why carry out this type of study. The methodology is well described and each of the analysis carried out is addressed, as are the results, in this context the discussion is discussed in a timely manner. However, some comments to be addressed and clarified in the manuscript are described in more detail below.

Author’s response: This comment raises three specific issues in the manuscript.

1. The introductory part … is specifically missing to describe other variables that also limit the distribution of birds.

Author’s response: I have addressed this later in the Introduction in two sections. In paragraph 4 of the Introduction, I have included the other variables likely to affect granivore distributions in general, by adding the following sentence (new or edited text underlined).

“Geological influence on granivore abundance is likely to be obscured by other bottom-up (e.g. climate, water-availability, vegetation structure, nest sites) and top-down (e.g. predation, disease) processes, disturbance (e.g. fire, flood) pressure from predators, and competition from other granivorous species [25, 26], and as well as the ability of species to switch between food sources in response to changes in seed availability [27, 28].” {L72-76 of the Revised Manuscript with Track Changes}

In paragraph 6 of the Introduction, I have added specific factors that influence the glossy black-cockatoo.

“The cockatoos also require hollow-bearing eucalypts in which to nest [32] and daily access to water [33]. Their distribution is therefore confined to areas of the Australian mainland where feeding and nesting habitat coincide and standing freshwater is available through the year, namely in subcoastal environments between South Australia and north east Queensland [34, 35]. However, not all areas that meet these criteria contain cockatoos, and the patchy distribution of The species has long perplexed ornithologists. The species is long-lived [36], largely disease-free [37] and – before clearance for agriculture fragmented the habitat – experienced minimal predation pressure or competition for nest hollows [38]. Gaps in the expected distribution may therefore result from nutritional limitations. Not all casuarina cones produce enough kernel matter to sustain the cockatoos, and the birds will reject trees that provide a suboptimal dry matter intake rate [31, 39]. Recent work has also suggested that they may also discriminate on seed nutrient content [40].” {L87-98}

2. The introductory part … is specifically missing … to describe the process of co-evolution or adaptation of the bird in depending strictly on that food resource.

I have now included information on the evolution of The species in paragraph 4 of “The species”.

“Calyptorhynchinae cockatoos have strong bills that allow them to extract seed from woody cones. While most species are arboreal foragers, and several eat casuarina seeds in small quantities [35], only the Glossy black-cockatoo has specialised to the extent of feeding almost exclusively on a single food source – the kernels of casuarinas [29]. This specialisation evolved around 7 million years ago [51, 54] as aridity developed across the Australian continent and casuarina woodlands expanded [55] It was possible because casuarina kernels meet the full nutritional needs of the cockatoos [31], to the extent that the birds spend only 26% of their time feeding in the non-breeding season and 36% in the breeding season [30].” {L175-182}

3. The problem statement must be highlighted in a better way, that is, the problem behind it and therefore why carry out this type of study.

Author’s response: The edits to paragraph 6 of the Introduction described above set the foundation for a clear problem statement. They describe that The species does not occur in all places where it could be expected if The species’ requirements for feeding habitat, nest sites and water are met, given that The species is long-lived and has few natural predators or diseases, but is known to be particular about the trees it feeds on. This leads to the problem statement at the end of paragraph 6 of the introduction:

“As seed production and nutritional composition is influenced by nutrient uptake from the soil, and therefore by the parent rock [41], I propose that the distribution of glossy-black cockatoos is also influenced by geology.” {L98-103}

Reviewer #1: Line 29-33. Although nutrients are essential, other biotic and abiotic variables are important and also contribute to the formation of rocks. How this type of geology originates historically and what time scale occurs in them to form.

Author’s response: I have addressed this by providing a more detailed description of soil formation in paragraph 1 of the introduction, as follows:

“All biological systems are nutrient-dependent [1]. In terrestrial ecosystems, most nutrients are ultimately derived from the parent rocks [2], although nitrogen (N) may also be derived from the atmosphere through nitrogen-fixing symbiotic microorganisms [3], and organic carbon (C) from plants and soil biota [4]. The most fertile soils are found on clay-rich rocks, such as basalt, limestone and fine-grained sedimentary rocks [5]. In addition, weathering (the product of climate, topography and biological activity) initially makes nutrients available, and – over time – leaches them from the soil profile. By changing the soil’s chemistry, texture, and pH, weathering affects the ability of the plant to take up nutrients, including by influencing the efficacy of N-fixation [3]. Hence, plant productivity tends to be limited by nitrogen on young soils, and by phosphorus (P) on ancient soils [6]. These limitations flow on to the animal populations that can be supported by the vegetation, which may be additionally limited by calcium (Ca) needed for bone and egg formation [7-9]. Hence, rocks – and the soils derived from them – have the capacity to control the distribution and productivity of plants and, thereby, of the animals that are dependent upon their seeds, fruits, flowers and leaves [10].” {L35-48}

Reviewer #1: L.56-59. What is the structure of the seeds and will there be a mutualistic relationship between food and bird?

Author’s response: I have provided details on the structure of the seed in the paragraph 2 of “The species” section of the Methods.

“Casuarinas produce woody cones, each with up to 100 seeds [31]. The seed has a single kernel and a winged seed coat (samara), which aids dispersal on cone maturity (in *Casuarina*), or after fire or stem death (in *Allocasuarina*) [46], although the majority of seeds fall below the canopy [47, 48].” {L151-154}

I have described the lack of benefits to the casuarinas in paragraph 5 of “The species”.

“Each cockatoo feeds on an average of 4-5 trees per day, taking an average of 61-78 cones per day in the non-breeding season, and 123-128 cones per day in the breeding season [30]. It systematically shreds each cone to extract the seeds one by one, neatly bisecting each in its bill to remove and ingest the single kernel [31]. The casuarinas gain little benefit from this exploitation, as the cockatoos usually extract and consume all viable seeds from each cone they handle [31], though occasionally drop half-consumed cones that still contain seeds [30].” {L80‑86}

Reviewer #1: L 60-62. Address the area of distribution of The species, how environmental factors influence the presence and distribution of The species.

Author’s response: The changes in paragraph 6 of the Introduction described above provide this information. {L87-103}

Reviewer #1: L 87-88. Add the compass rose to the figure, that is, the orientation.

Author’s response: Added to Fig 1 as requested.

Reviewer #1: L 89-92. The information is very extensive, we suggest synthesizing more.

Author’s response: Caption to Fig 1 condensed as requested. {L128-132}

Reviewer #1: L 98. It is not clear, is it a bush or a tree? In what geological situations is one and the other supported.

Author’s response: I have revised paragraph 1 of “The species” to clarify The species’ habit, and provide the geological and soil settings, as follows:

“Drooping sheoak is a shrub to small tree that forms monospecific stands of low woodland/tall shrubland on coastal sands or shallow soils over sandstone, limestone or basalt [44, 45]. It also occurs as an understorey species in open forest on acidic gradational soils over sandstone or metasandstone, frequently in association with sugar gum (*Eucalyptus cladocalyx* F. Muell.) or other eucalypts [44, 45]... Black sheoak (a shrub to small tree) grows mainly on poor sandy, skeletal or rocky soils [44, 45]. Forest oak (a medium-sized tree) is found on deeper, more fertile soils that overlay basalts and a range of igneous and sedimentary rock types [44, 45]. Both grow in the understorey of eucalypt-dominated open-forests and tall open forests in eastern Australia, although black sheoak also forms monospecific stands on rocky granitic outcrops [44].” {L140-149}

Reviewer #1: L 105-110. By presenting these characteristics in Casuarinas, as is the effect for other plant species that grow in the soil.

Author’s response: I have clarified this by describing the process in general in paragraph 2 of “The species”, along with how Casuarinas differ from other plants.

“In most plants, these nutrients are derived from the soil, often with the aid of symbiotic organisms [2, 6]. Casuarinas belong to a suite of non-leguminous plants that derive N from the atmosphere through its symbiotic relationship with the N-fixing actinorhizal bacterium Frankia Brunckhorst 1885 [49], which is influenced by soil chemistry [3]. Their acquisition of other nutrients is enhanced through endomycorrhizal and/or ectomycorrhizal associations and cluster root formations [50].” {L155-163}

Reviewer #1: L 111-112. Briefly describe the characteristics of The species.

Author’s response: In addition to the information already provided on The species’ breeding and feeding behavious, I have provided additional information on the cockatoo’s characteristics in paragraph 3 of “The species”.

“The glossy black-cockatoo is a small (400-520 g) member of Australia’s endemic Calyptorhynchinae cockatoos [32, 51]. The males are black with red windows in the tail. The females are similar, but each has a unique combination of yellow patches on the face and neck, and the red windows are interspersed with black bars.” {L164-167}

I have also included further information in paragraph 4 of “The species”.

“Calyptorhynchinae cockatoos have strong bills that allow them to extract seed from woody cones. While most species are arboreal foragers, and several eat casuarina seeds in small quantities [34], only the glossy black-cockatoo has specialised to the extent of feeding almost exclusively on a single food source – the kernels of casuarinas [29].” {L175-178}

Reviewer #1: L 118. What historical evolutionary process or other factor caused The species to depend on this food resource.

I have provided this information in paragraph 4 of “The species”.

“This specialisation evolved around 7 million years ago [51, 54] as aridity developed across the Australian continent and casuarina woodlands expanded [55]. It was possible because casuarina kernels meet the full nutritional needs of the cockatoos [31], to the extent that the birds spend only 26% of their time feeding in the non-breeding season and 36% in the breeding season [30].” {L178-182}

Reviewer #1: L 134-136. This is confusing. It is indicated that there is a study as background that provides the opportunity to evaluate the variables in seeds and geology. Clarify this wording.

Author’s response: I have provided clarification as requested.

“The studies reported here reassessed the influence of seed variables on tree selection by the cockatoos (study 1), and examined the influence of geology and soils on these controlling variable(s) (study 2). They used seed samples from a dataset that had been produced for an earlier publication that examined drooping sheoak tree selection by glossy black-cockatoos on Kangaroo Island [31]. As well as the 76 samples used in the earlier publication, this dataset also included additional samples from Kangaroo Island, as well as samples from the Riverina and mainland South Australia. All samples were collected between January 1996 and August 1997.” {L200-L210}

Reviewer #1: L 144-153. Argue if the samples used are sufficient, because, based on whom and what the bias would be or not.

Author’s response: I have provided a robust justification for the sample protocol. Given that this is standard and tested protocol, discussion of bias seems unnecessary.

“Each cone sample was a minimum of 10 mature (red to brown) cones from the current year’s crop and was dried at 60°C to constant weight, following Clout (39), a procedure that has been adopted by subsequent authors [31, 58], [though sometimes with smaller sample sizes; 40]. In each of these publications, this protocol has been shown to successfully distinguish between feeding and non-feeding trees.” {L216-220}

Reviewer #1: L 168-170. What is the bias or not in selecting the samples in that way, argue.

Author’s response: Explanation provided as requested.

“To avoid the impact of non-independent samples [67], where duplicate samples were available from an individual tree, a single sample was randomly selected for inclusion in the analysis.” {L244-247}

Reviewer #1: L 235-241. What software was used to make these projections?

Author’s response: Information provided as requested.

“Both datasets were filtered using information provided in the original datasets to exclude records with a spatial accuracy of more than 2 km, as well as those missing key spatial or temporal collection data, or that were of cultivated plants, and retaining only those records from within New South Wales, as determined using Quantum GIS [QGIS; 43] (S4 Table).” {L321-326}

Reviewer #1: L 260-261. Support why this model was selected as residual.

Author’s response: References given to justify model selection methods.

“The preferred model was selected based on minimum residual deviance [83], followed by Akaike Information Criterion [AIC; 84], and lastly the Bayesian Information Criterion [BIC; 85].” {L347-349}

Reviewer #1: L 279-283. Synthesizing the information is very extensive.

Author’s response: Caption to Fig 2 condensed as requested. {L369-376}

Reviewer #1: L 342-350. Synthesize the information, be more specific.

Author’s response: Caption to Fig 3 condensed as requested. {L436-454}

Reviewer #1: L 377-383. Synthesize the information.

Author’s response: Caption to Fig 4 condensed as requested. {L483-491}

I have similarly condensed the caption to Fig 5. {L520-524}

L 464-471. Because it is assumed that this would have been achieved, sustain.

Author’s response: I have condensed and clarified this section of text, making more explicit reference to the study of North et al., in which false conclusions were made based on whole seed, and cautioned against doing so.

“As no nutrient was found to be higher in the kernels of feeding trees than in those of non-feeding trees, the conclusion by North *et al.* (40) that the cockatoos select feeding trees based on specific nutrients is not supported. Their study analysed whole seed, which the cockatoos do not eat. The current study shows that any nutrient differences between the whole seed of feeding and non-feeding trees are simply a product of the differences in the composition of the kernels and the samara. As feeding trees have more kernel matter per cone (higher Food Value) than those of non-feeding trees, their whole seed is likely to have elevated levels of nutrients that are concentrated in the kernels (P, N, S, Mg, K and Mn, this study; fatty acids [31]), and depressed levels of nutrients that are concentrated in the samara (Ca, Na, Fe and Cu, this study; ash, fibre and carbohydrates [31]). Had the current study considered the composition of whole seed alone, as was done by North *et al.* (40), it might have erroneously concluded that the cockatoos were selecting feeding trees to minimise the intake of Cu and Zn, or to maximise N and Mn intake. Hence, I caution against making conclusions about dietary preferences of species that only consume the kernels based on whole seed.” {L577-600}

Reviewer #2: the article was very well thought out and written, I congratulate the authors for their effort in carrying out the analyzes and creating a well-done text.

I only have a few suggestions:

Author’s response: Responses to these detailed comments are addressed below.

Reviewer #2: Line 45: "in several species in Australia [9, 10] and Africa [11] and North America [12]." add "," ; "in several species in Australia [9, 10], Africa [11] and North America [12]."

Author’s response: Corrected as requested. {L59-61}

Reviewer #2: Line 436: here you could explain more why nitrogen influences animal abundance.

Author’s response: Detail provided as requested.

“N is considered to be the dietary nutrient most likely to be limiting to granivores [91, 92]. It is required for the production of amino acids, proteins, and other essential compounds, including DNA, RNA, vitamins and hormones [92]. Parrots require a dietary intake of 11-20% protein [93], which is essential for the production and maintenance of muscles and feathers [92, 93]. The protein content of seeds can vary between 3% and 48% [94], so active selection of seeds based on N content could be expected.” {L549-554}

Reviewer #2: Line 441: what are these foods preferred by Amazon parrots? what types of seeds?

Author’s response: I have provided the information that the measurements were based on 29 species of seed. This is too many to name individually. I have also slightly revised this section to follow on from the discussion about nitrogen, as follows:

“However, as the protein concentration of drooping sheoak kernels (44.9 ± 0.4%) is far higher than that required by parrots, glossy black-cockatoos do not need to seek out the most nitrogen-rich kernels. This should also be the case for cockatoos feeding on other casuarinas, with the whole seed of black sheoak containing 27.1 ± 3.1% protein [39] and those of slaty sheoak (*A. muelleriana*) containing 31.6 % protein [95] (with the levels in the kernels expected to be much higher), or for Amazonian parrots feeding on the whole seed of a range of species whose protein content averaged 21.9% [averaged across 29 seed species and 17 parrot species; 96].” {L554-L565}
